# Supplementary material for: Microbiome time series data reveal predictable patterns of change
Source: Microbiol Spectr. 2024 Aug 20;12(10):e04109-23. doi: 10.1128/spectrum.04109-23 (PMC11448390; doi:10.1128/spectrum.04109-23)
Supplement: Supplemental material — Fig. 1 to 23; Table S1. [file spectrum.04109-23-s0001.pdf]

Microbiome time series data reveal predictable patterns of change

Supplementary information

Zuzanna Karwowska[1,2], Paweł Szczerbiak[1], Tomasz Kosciolek[1,3*]

1 Malopolska Centre of Biotechnology, Jagiellonian University, Krakow, Poland
2 Doctoral School of Exact and Natural Sciences, Jagiellonian University, Krakow, Poland
3 Dept. of Data Science and Engineering, Silesian University of Technology, Gliwice, Poland
* *tomasz.kosciolek@polsl.pl*

Table of contents

Whole community analysis 2
Individual features analysis 8
Cluster analysis 13
- NetworkX graphs 13
- Regime evolution in time 20
- Examples 23
- Sanity check - Euclidean distance vs |ρ| 24
- Hierarchical clustering 25

Input data processing 28

Whole community analysis

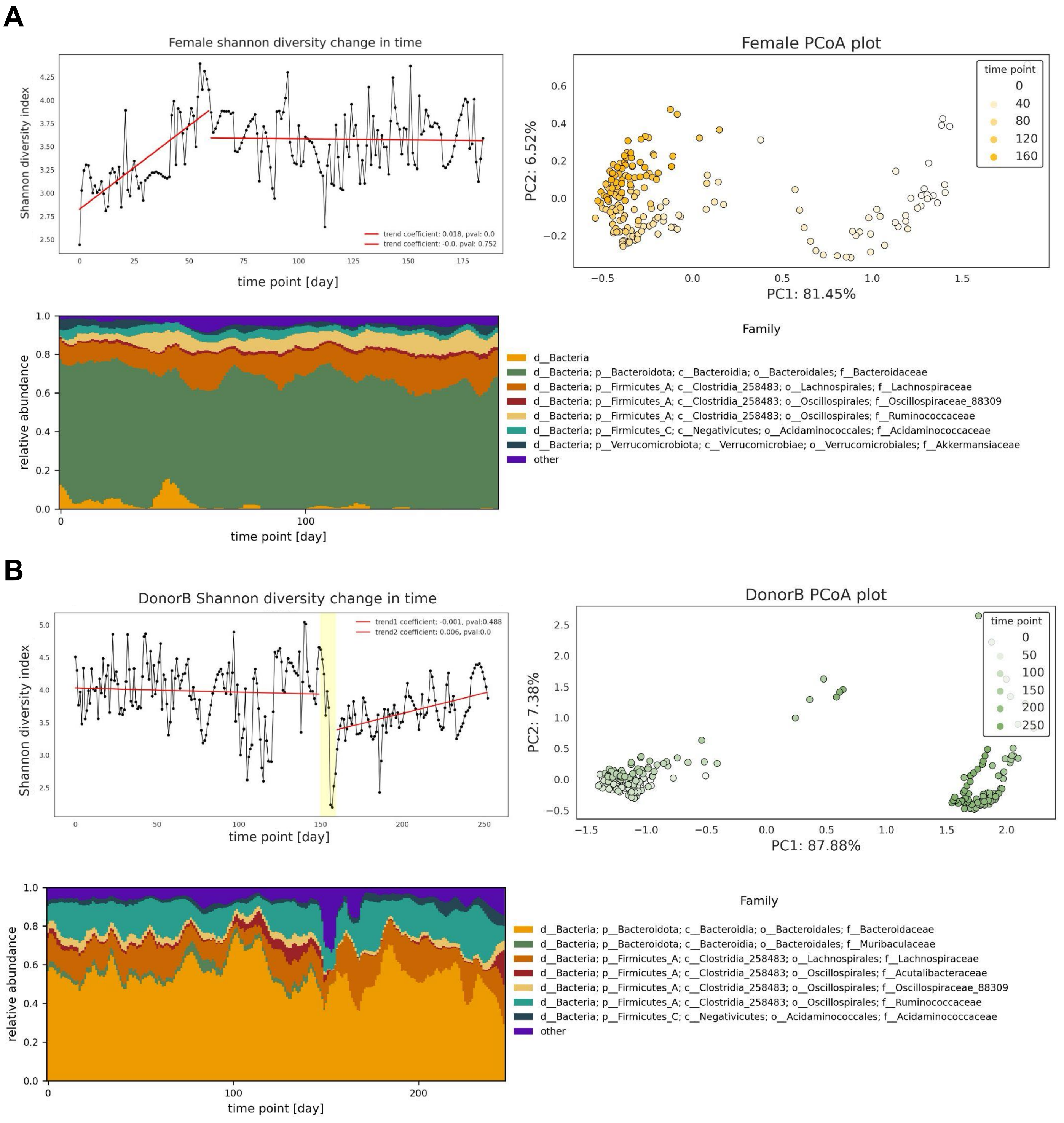

Supplementary Figure 1. Overview of microbiome behavior in time for female (A) and the donorB subject (B). The female subject trend is growing, however, it can be seen from the PCoA analysis that the first 50 days are different from the rest of the time series and that the trend starting from day 50 is stable. From donorB metadata we know that he was suffering from diarrhea on days around day 150. We see that this event decreases his alpha diversity which drops around day 150 and then increases to the baseline level. Also during the event, we can see changes in taxonomy as new taxa appear.

Shannon diversity index **Faith's phylogenetic index**

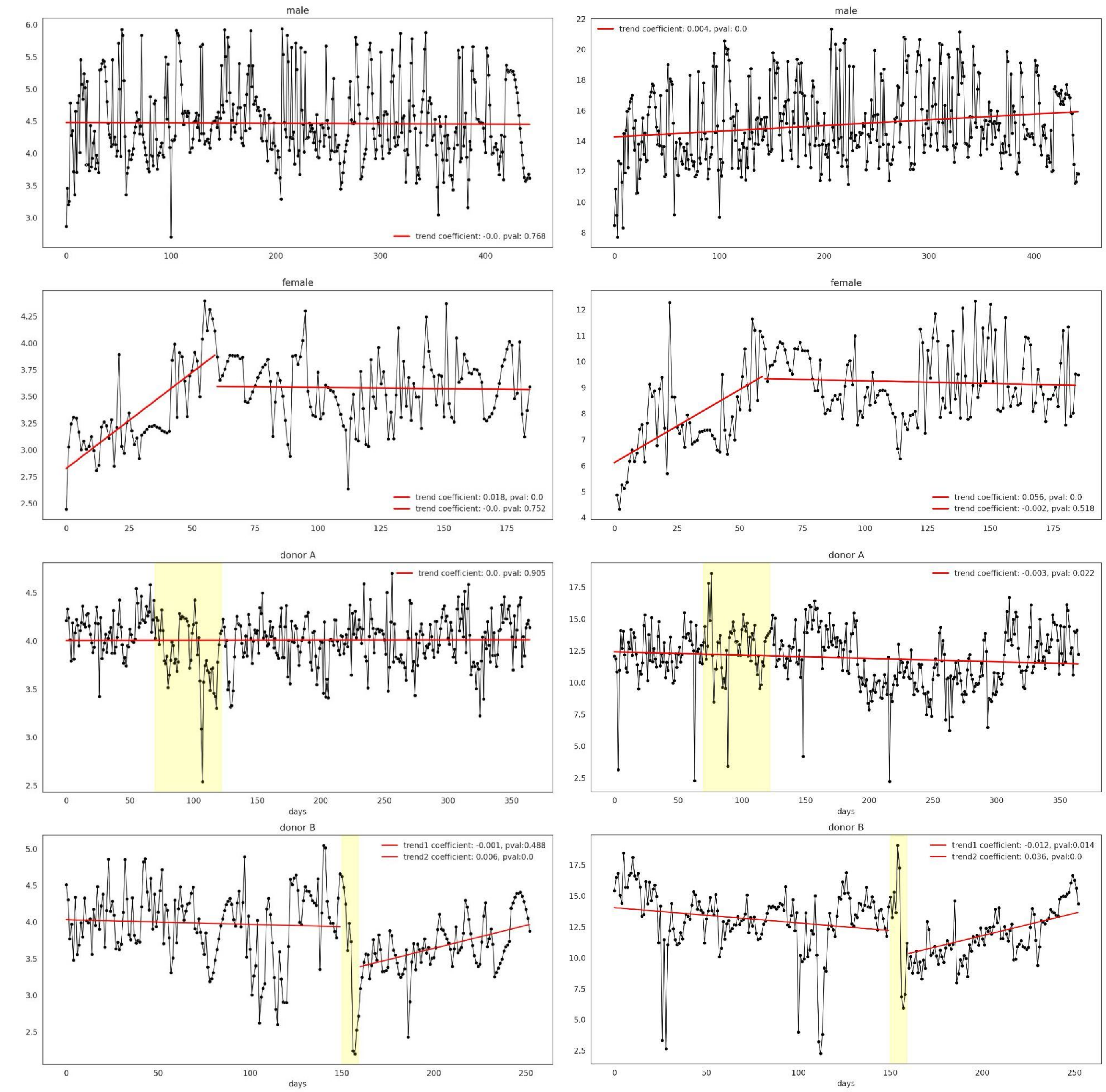

Supplementary Figure 2. Shannon diversity index and Faith's phylogenetic index behavior in time (each row represents one subject). Red lines represent the trend and trend coefficients calculated using regression analysis. Male alpha diversity remains relatively stable over time. In the female subject, the trend stabilizes from day 50 onward. Donor A's alpha diversity is stable over time despite perturbations. Donor B's alpha diversity drops significantly around day 150 and then gradually returns to baseline.

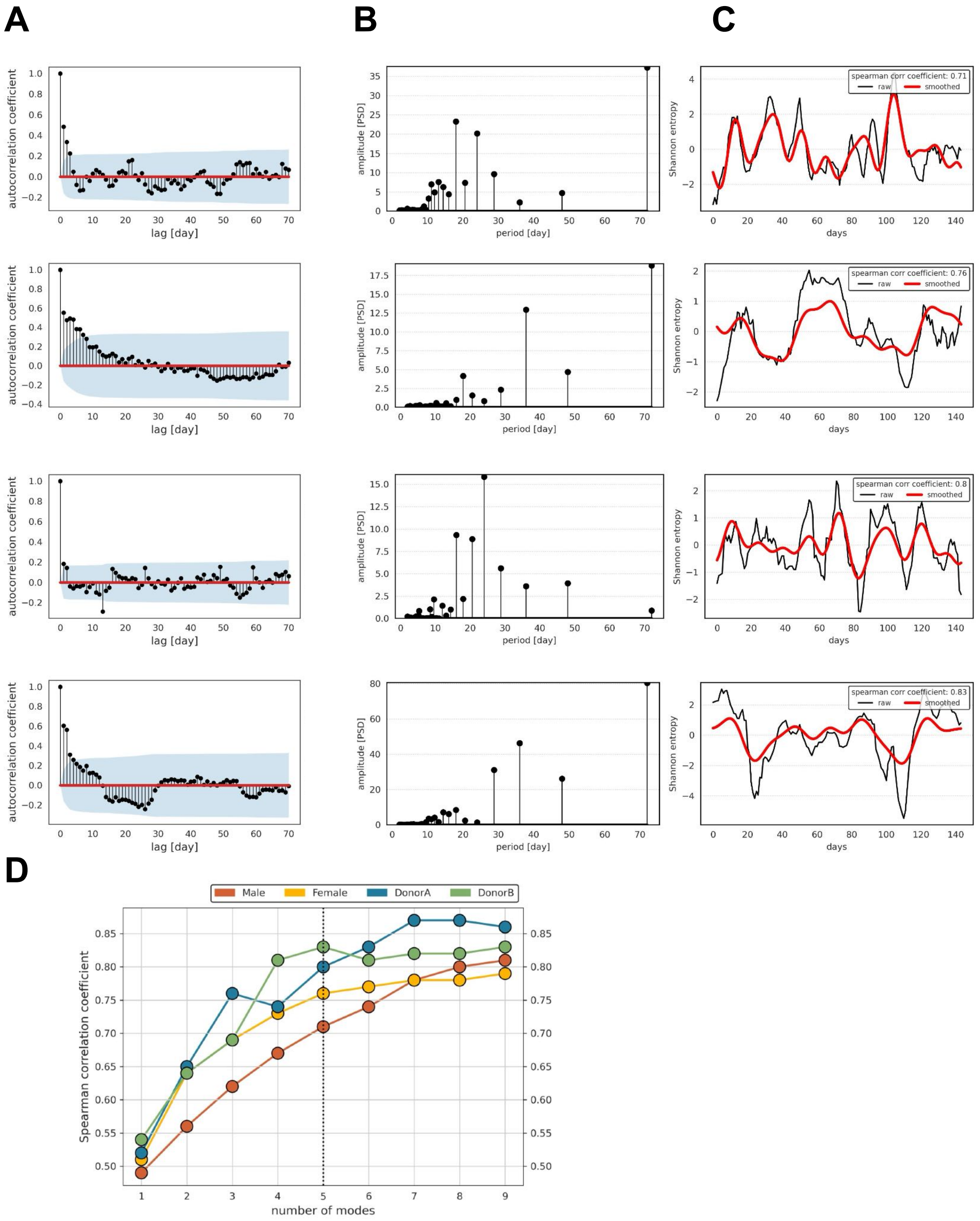

Supplementary Figure 3. Faith's phylogenetic index behavior in time. A. Autocorrelation coefficients plots (the blue area indicates the significance level, representing confidence intervals for the autocorrelation coefficient). **B.** Spectrogram showing most dominant seasonalities of human gut microbiome. **C.** Reconstruction of alpha diversity using 5 dominant seasonalities plotted against raw alpha diversity change in time. **D.** Plot showing the relationship between number of used seasonalities to reconstruct alpha diversity and the seasonal reconstruction score.

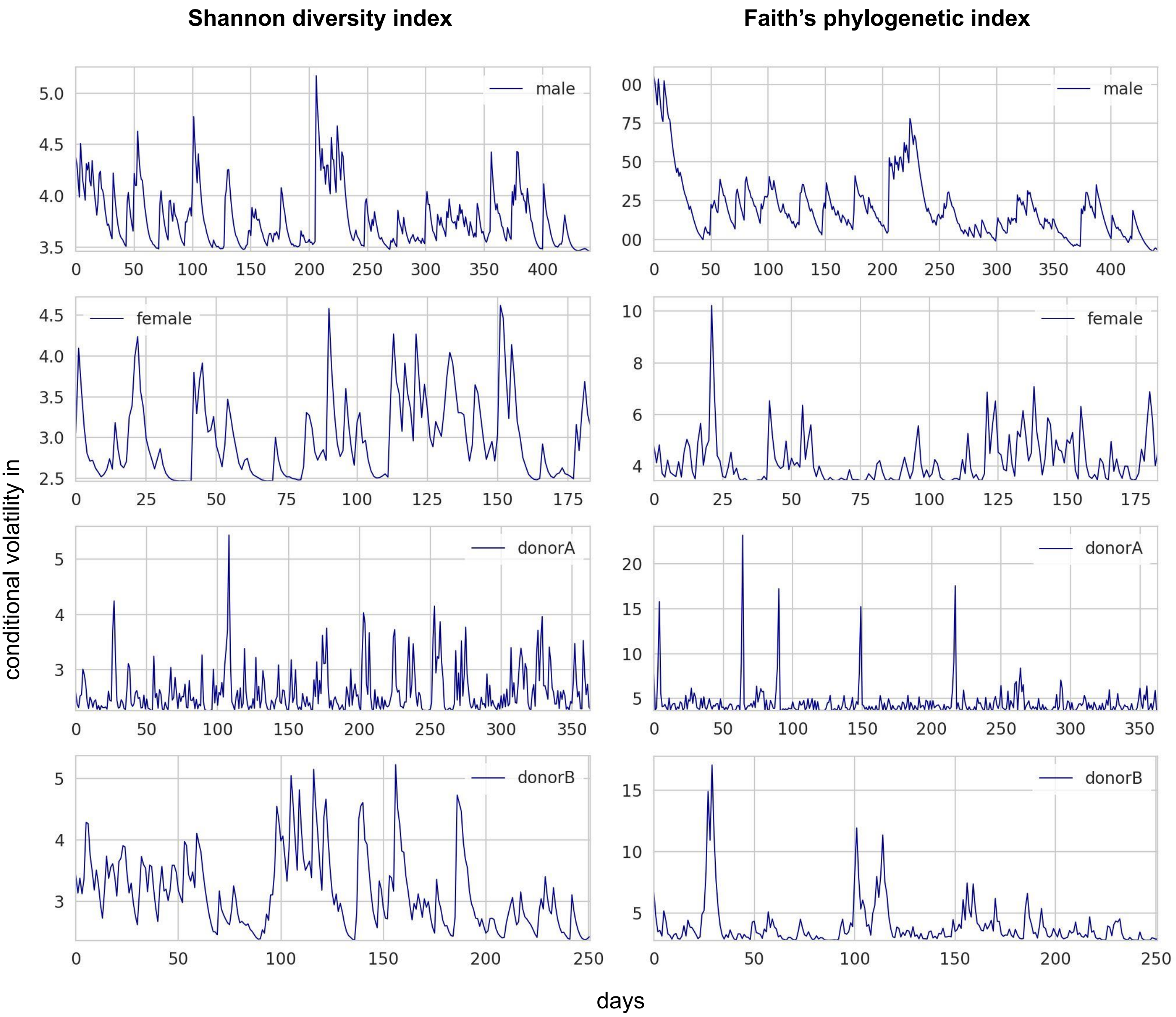

Supplementary Figure 4. Line plots of conditional variance estimated using GARCH model showing higher variance periods in each alpha diversity time series. Left: Shannon diversity index volatility; right: Faith's PD index volatility.

A

Subject	p	d	q	N modes	Mode’s period [days]
male	3	0	10	3	20; 10; 5.7
female	2	0	7	6	11.4; 7.2; 4.4; 5; 3.6; 3.3
donorA	3	0	1	6	23.3; 14; 7; 5.8; 3.1; 3.5
donorB	4	0	7	6	10; 26.7; 6.1; 7.2; 4.4; 5.3

B

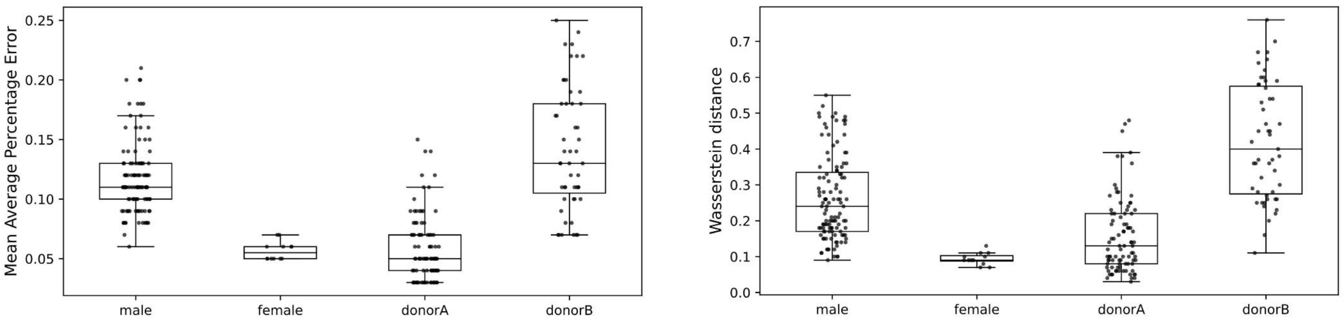

Supplementary Figure 5. Dynamic Arimax model results. A. Parameters for an ARIMAX model, determined through k-fold cross-validation for each subject. The parameters include: **p** - the number of lag observations included in the model, or the order of the autoregressive (AR) part; **d** the degree of differencing required to make the time series stationary. q - the size of the moving average (MA) window, indicating the number of lagged forecast errors in the prediction equation. Additionally, the figure outlines the exogenous variable derived from FFT analysis, indicating; **N Modes -** the number of significant frequency modes identified; **Mode's Period** - the periods associated with each mode, ordered by their amplitude from highest to lowest, used to construct the seasonal exogenous variables of the ARIMAX model. **B.** The test set cross-validation results of the predictive model performance are presented, with the error measure calculated on a 20-day interval every 5 days. The first set of boxplots displays the mean average percentage error for each fold, while the second set demonstrates the Wasserstein distance. The analysis reveals that the model's performance is not consistently favorable across all folds. There are instances where the model does not perform well, indicating a lack of generalizability in its predictions. This lack of generalizability is attributed to external changes, such as events occurring in donorA and donorB. These findings suggest that the predictive model may be influenced by unpredictable factors or events specific to individual donors, making it challenging to achieve consistent and accurate predictions. Further investigation and refinement of the model may be necessary to enhance its generalizability and robustness to external variations.

Individual features analysis

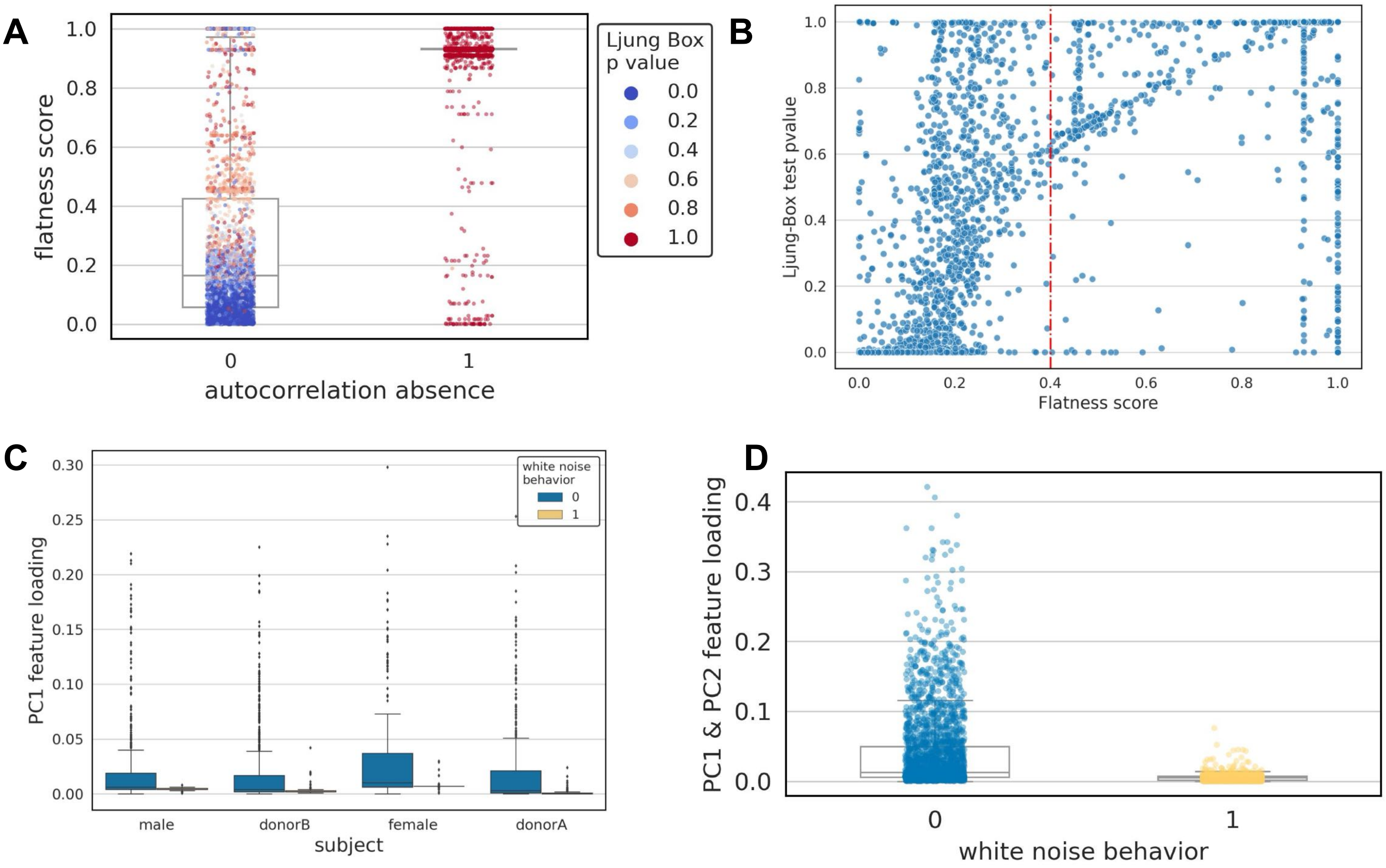

Supplementary Figure 6. White Noise Definition. A. and **B.** display the distribution of Ljung-Box test p-values against the flatness score for each taxon analyzed. These figures serve to illustrate the relationship between these two measures. Based on the observed distribution, we have chosen to introduce a new artificial measure termed "white noise behavior." This measure encompasses taxons that meet the following criteria: a Ljung-Box p-value less than 0.5 and a flatness score above 0.4. However, we recommend conducting a thorough analysis of such a distribution on individual datasets to determine the optimal threshold for defining white noise behavior. **C.** and **D.** provide additional insight into the impact of features identified as noise on data variance. This figure demonstrates that these identified features have minimal or no effect on the overall variance of the data.

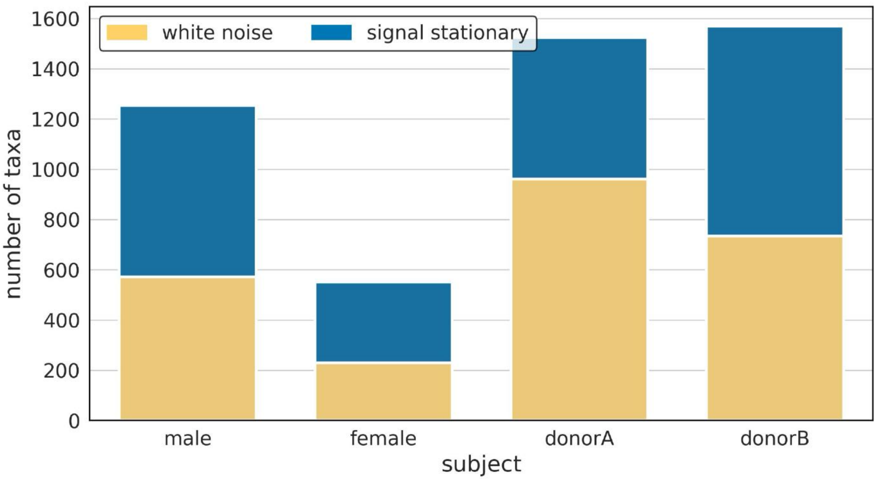

Supplementary Figure 7. Number of White noise bacteria in human gut microbiome.

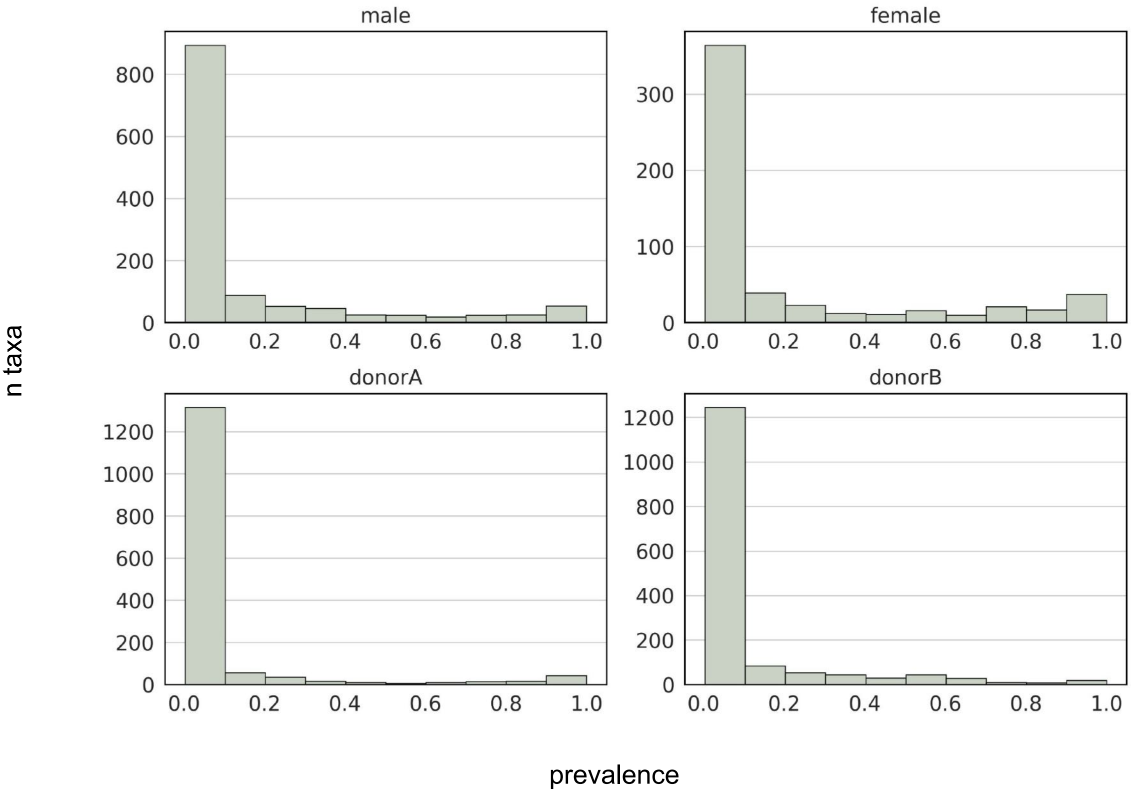

Supplementary Figure 8. Taxa prevalence between subjects. A significant proportion of bacteria were classified as rare or predominantly absent (they are present in less than 10% of time series), while a smaller fraction was consistently present in the gut microbiome. Another subset appeared intermittently, making them neither rare nor constantly present. Surprisingly, almost half of each individual's microbiome was classified as noise, likely resulting from technical factors and metabolic conditions.

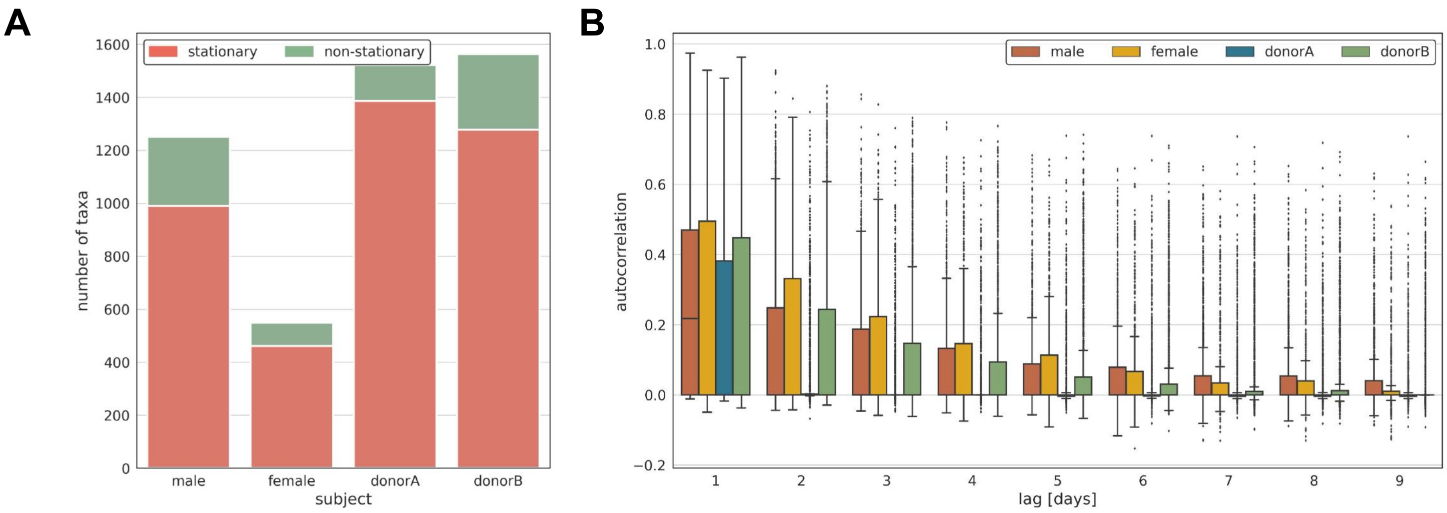

Supplementary Figure 9. A. Number of stationary and nonstationary taxa in foursubjects; **B.** Taxa autocorrelation.

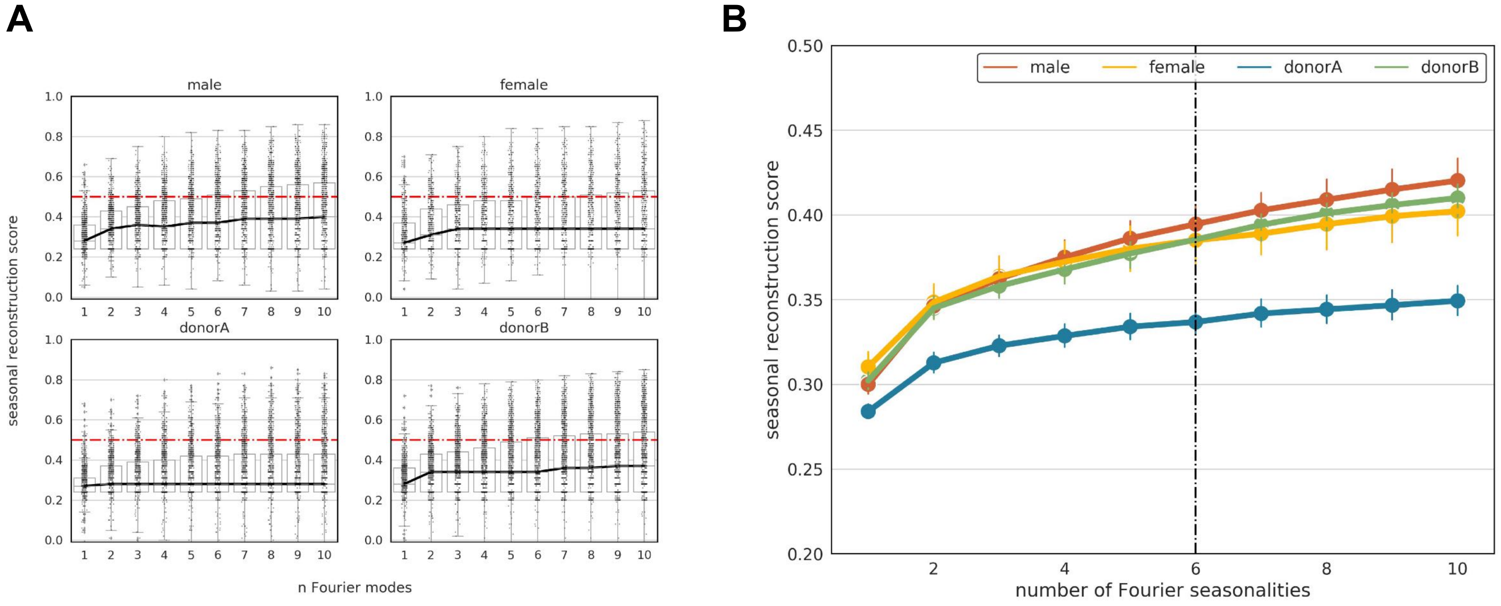

Supplementary Figure 10. Number of seasonalities present in human gut microbiome. A. The analysis of the required number of seasonalities needed to accurately reconstruct the raw bacterial fluctuation in time. Each data point represents a specific ASV. The x-axis represents the number of seasonalities required for reconstruction, while the y-axis depicts the reconstruction score. Each dot corresponds to the score for reconstructing each bacterial species. **B.** Mean relationship between the number of modes used for reconstructing raw bacteria counts and the corresponding reconstruction score. The x-axis represents the number of modes, whereas the y-axis denotes the mean seasonal reconstruction score. The results demonstrate that, in contrast to alpha diversity, different bacterial species exhibit diverse behaviors. Additionally, the analysis highlights that the number of Fourier modes needed to accurately reconstruct the raw signal varies significantly among bacterial species (Panel A). These findings shed light on the complexity and heterogeneity of the gut microbiome seasonal dynamics.

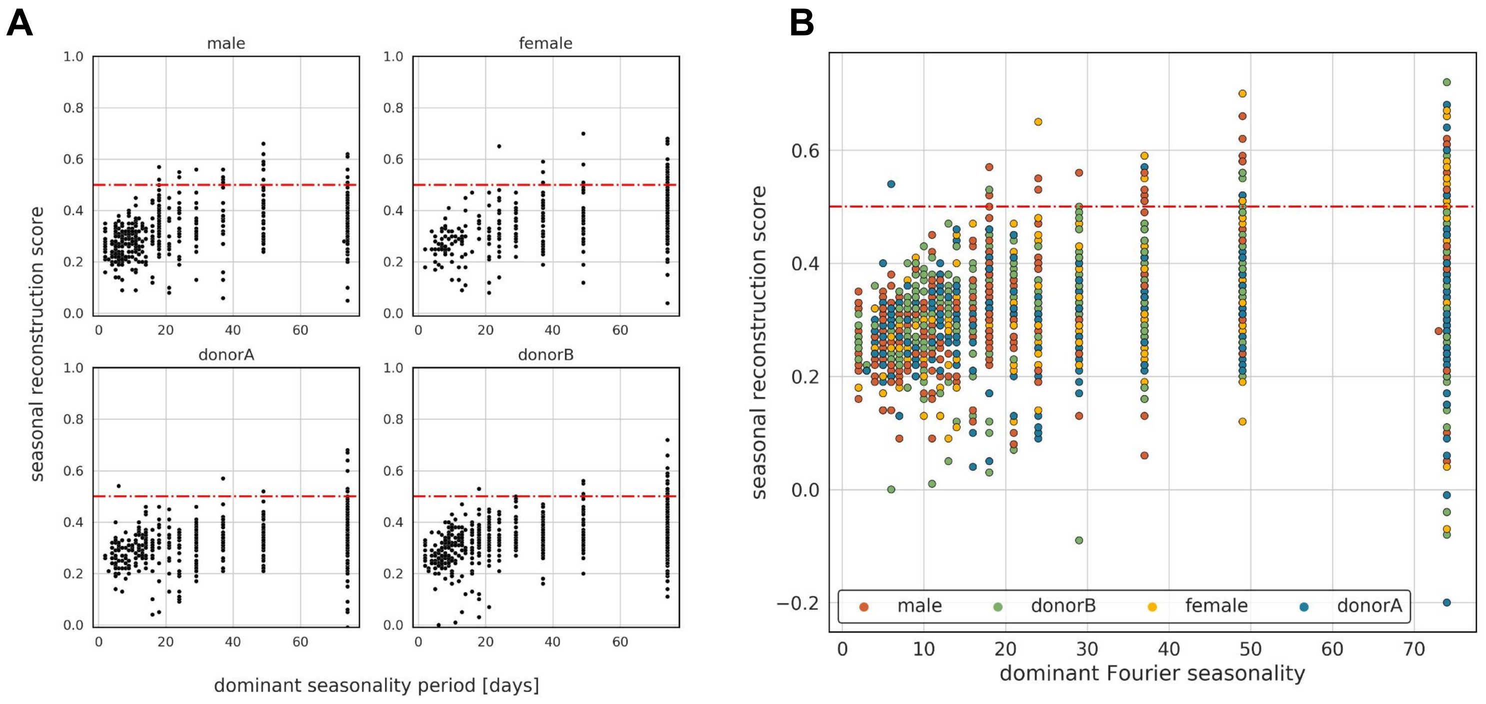

Supplementary Figure 11. Dominant seasonalities in human gut microbiome. A. Dominant bacterial seasonality and corresponding seasonal reconstruction score. The red line represents a score cutoff of 0.5. It is observed that several bacteria exhibit short seasonalities identified by FFT with low reconstruction scores, suggesting that they may be noise. Moreover, longer seasonalities tend to have higher reconstruction scores. **B**. The panel demonstrates that the seasonalities in the gut microbiome are generally similar across analysed subjects, indicating consistency in the observed patterns. Overall, the plot provides insights into the dominant seasonalities present in the human gut microbiome. The findings suggest the presence of meaningful seasonal patterns in certain bacteria, while cautioning against short seasonalities with low reconstruction scores. Additionally, the consistent patterns observed across subjects contribute to our understanding of the general characteristics of gut microbiome seasonal dynamics.

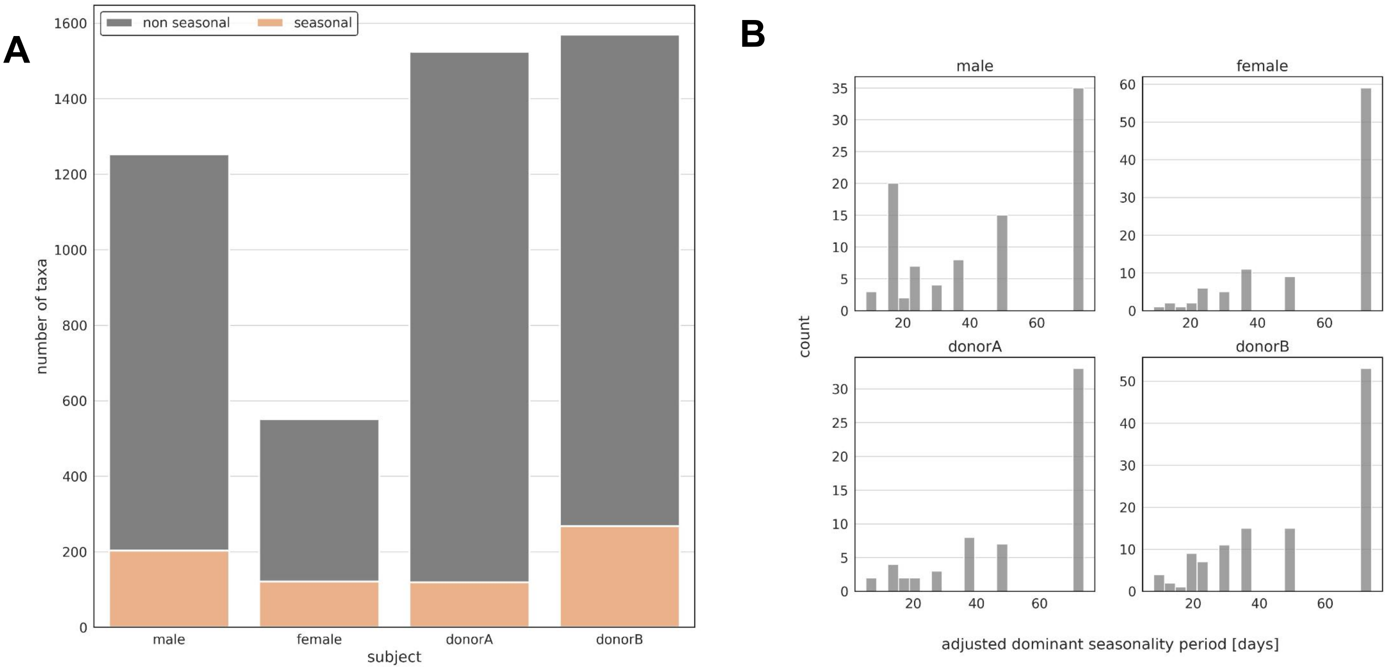

Supplementary Figure 12. Number of seasonal bacteria in human gut microbiome. A. Number of ASVs classified as seasonal in each subject. The classification is based on the successful reconstruction of bacterial raw counts using 5 Fourier seasonalities and a minimum seasonal reconstruction score of 0.5. This panel provides an overview of the prevalence of seasonal bacteria across subjects. **B.** Adjusted dominant seasonalities in bacteria for each subject. The adjusted seasonalities were identified based on a reconstruction correlation score of at least 0.5. This panel highlights the presence of more reliable and significant seasonal patterns in the bacterial population of each subject.

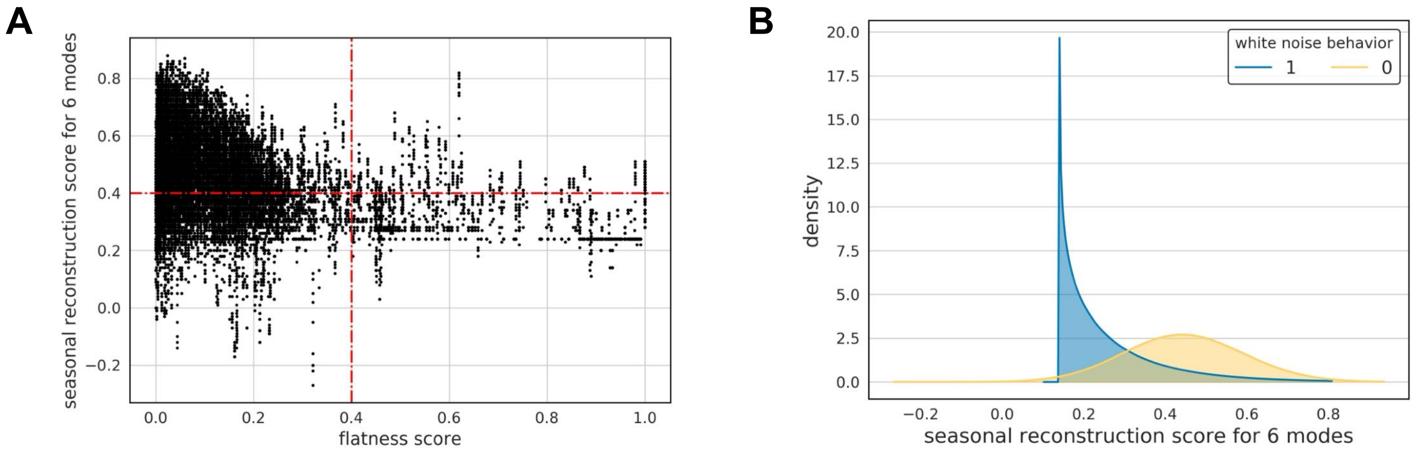

Supplementary Figure 13. Different Characteristics of Seasonal Bacteria in the Human Gut Microbiome. A. Relationship between the Seasonal Reconstruction Score of 5 Fourier Modes and Bacteria Flatness Score: This figure depicts the association between the seasonal reconstruction score, derived from the analysis of six dominant Fourier modes, and the bacteria flatness score. The results reveal that seasonal bacteria exhibit a remarkably low flatness score, indicating a high degree of regularity in their seasonal patterns. This observation serves as a sanity check, validating the presence of distinct seasonal behavior in these bacteria. **B.** Seasonal Reconstruction Score of 5 Fourier Modes for Bacteria Classified as White Noise: This panel showcases the seasonal reconstruction scores for individual bacteria, differentiating those classified as white noise from bacteria classified as ‘signal’. Notably, bacteria displaying stochastic behavior, characterized by white noise patterns, generally do not exhibit discernible seasonality. Despite considering their six dominant seasonalities, we are unable to accurately reconstruct the seasonal patterns of these bacteria. This finding underscores the absence of predictable seasonal behavior in bacteria with stochastic dynamics.

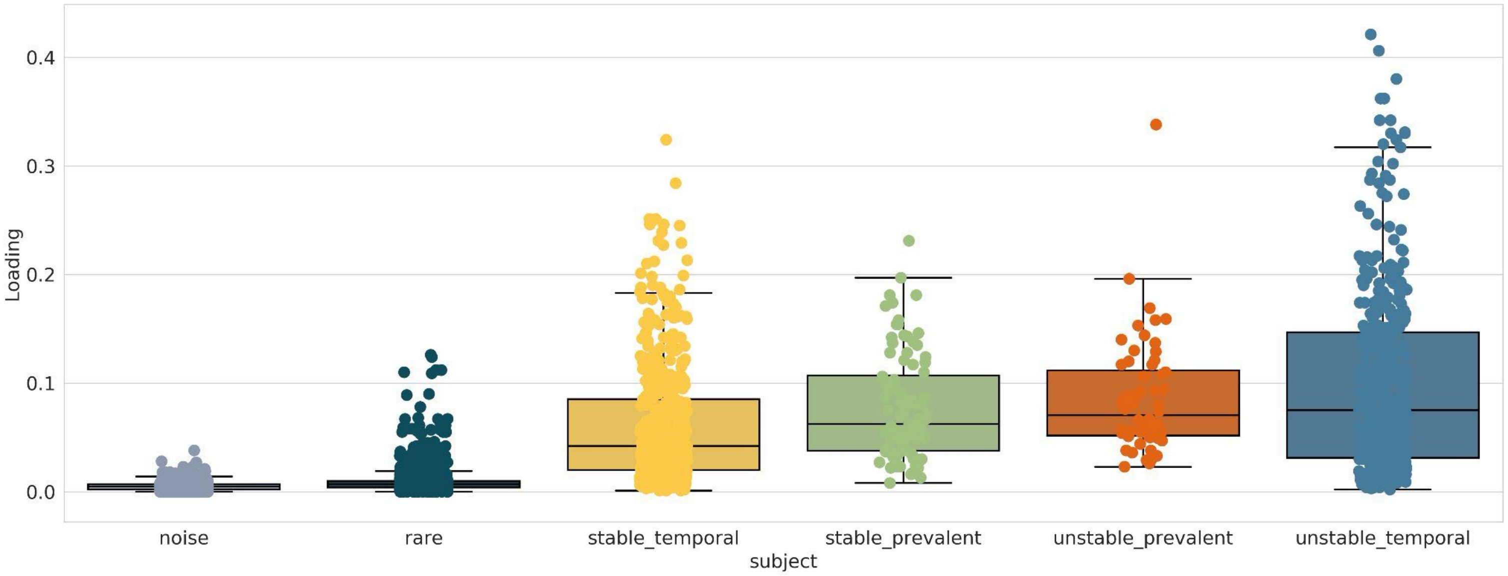

Supplementary Figure 14. Cumulative PCoA feature loading of each bacterial regime. Each point represents a ASV displaying different regime. We can see that the bacteria belonging to rare and noise regime has the lowest regime despite being the most numerous. On the other hand unstable and temporal regime has the highest loading. PCoA was calculated for each subject separately based on it's Aitchison distance matrix between timepoints.

Subject	All	Rarefied	Signal	Noise	Rare
male	1399	1253 (90%)	358 (26%)	426 (30%)	615 (44%)
female	591	551 (93%)	187 (32%)	189 (32%)	215 (37%)
donorA	2862	1524 (53%)	210 (7%)	643 (23%)	2009 (70%)
donorB	1524	1005 (66%)	280 (18%)	318 (21%)	926 (61%)

Supplementary Table 1. Number of bacteria in each subject. All percentages are computed with respect to "All" (second column)l. Signal bacteria (stable / unstable and prevalent / temporal - see Supplementary Figure 14) comprises ~30% of male and female microbiomes and 7 / 18% of donorA / donorB microbiomes respectively (this may be an artifact coming from experiment). However, the absolute number is stable for all subjects and ranges between 187 and 358.

Cluster analysis

NetworkX graphs

Panels in Supplementary Figure 15 (see caption in p. 20) corresponds to Fig. 5A in the main text but using all bacteria (subplot A), coloured by different features (B-K) or generated using different ρ_{thr} (L-M). Specifically, we can notice that ρ_{thr} = 0.6 provides better graph separability as compared to 0.5 or 0.7 (compare Fig. 5A and subplots L, M in Supplementary Figure 15). Medium-size subgraphs contain abundant species (subplots B, C) with complex dynamics (both stationary and non-stationary, seasonal and non-seasonal, and different occurrence percentage). On the other hand, the cloud is built from bacteria that are different to anything else and are probably the most intriguing. It also exhibits rich dynamics but this is more subject dependent. Seasonal bacteria (I, J) can be found in both regions. Moreover, similar number of seasonal species are present in all subjects' clouds. Most abundant species (with largest PC1+PC2 loading; subplots G, H) are present in the connected subgraphs but not all subgraphs comprise such species - many of them can also be found in the cloud. All regions exhibit high taxonomic diversity (E) but one can identify smaller clusters which are homogeneous. According to expectations, rare bacteria clusters are stationary (K). The same holds for abundant clusters but, interestingly, we can also find large connected components dominated by non-stationary species.

A

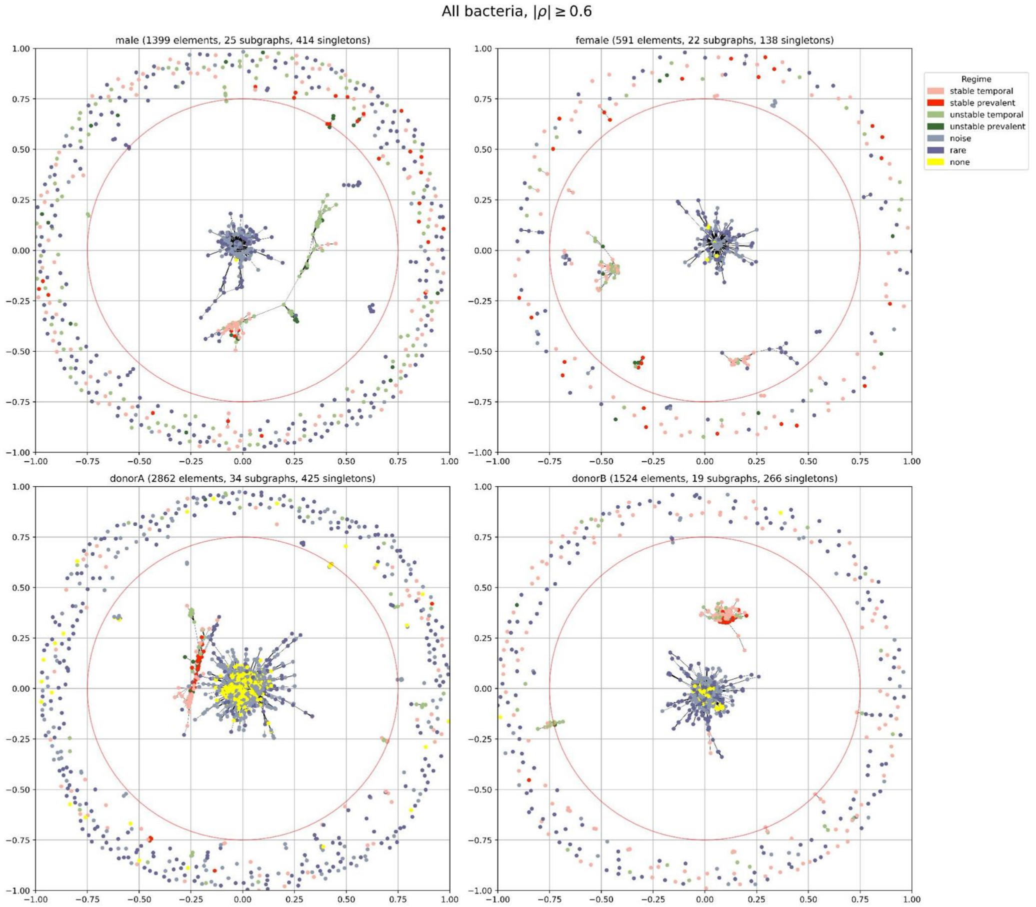

B

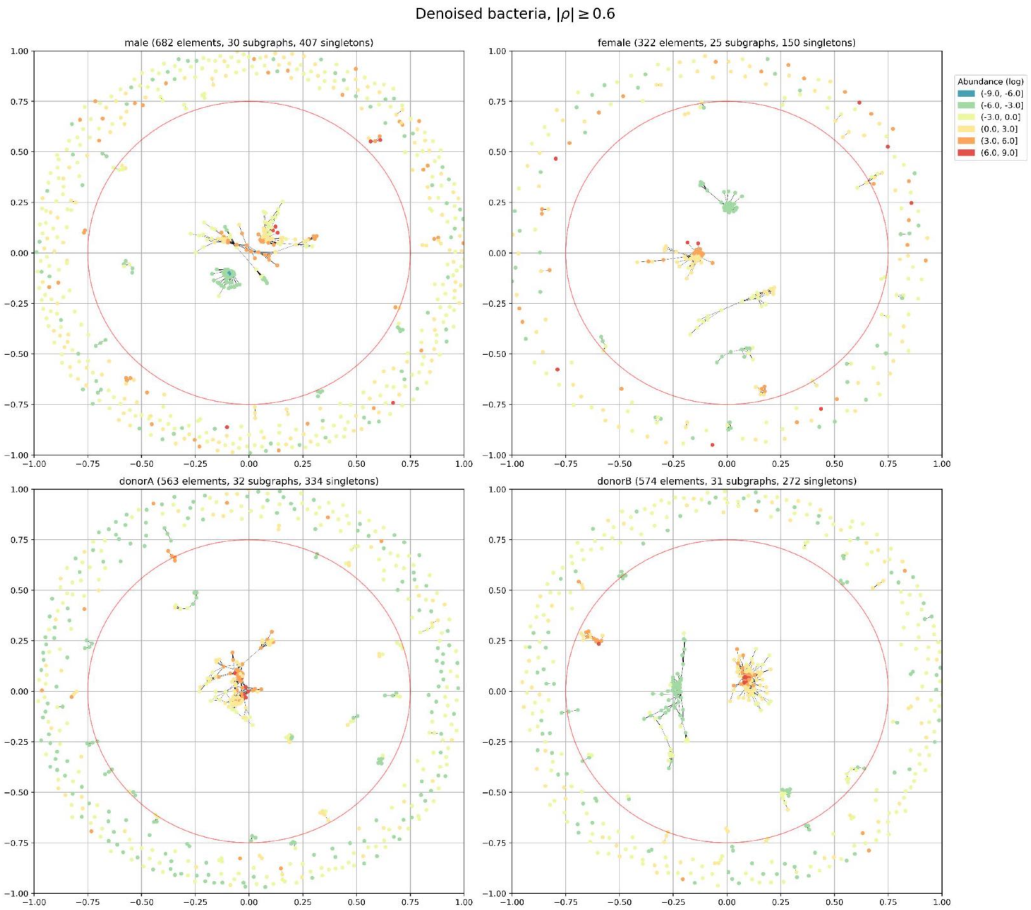

Denoised bacteria, |ρ| ≥ 0.6
male (682 elements, 30 subgraphs, 407 singletons)
female (322 elements, 25 subgraphs, 150 singletons)
donorA (563 elements, 32 subgraphs, 334 singletons)
donorB (574 elements, 31 subgraphs, 272 singletons)
Abundance (log)
(-9.0, -6.0]
(-6.0, -3.0]
(-3.0, 0.0]
(0.0, 3.0]
(3.0, 6.0]
(6.0, 9.0]

C

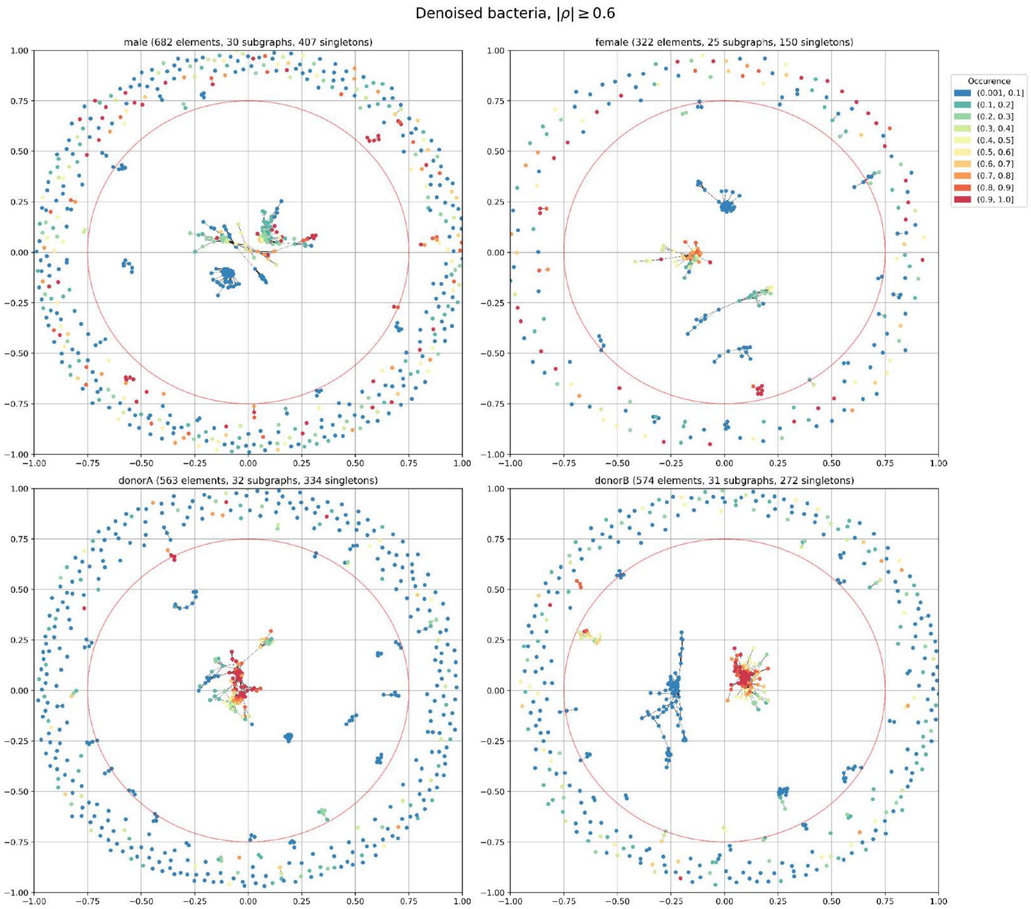

Denoised bacteria, |ρ| ≥ 0.6
male (682 elements, 30 subgraphs, 407 singletons)
female (322 elements, 25 subgraphs, 150 singletons)
donorA (563 elements, 32 subgraphs, 334 singletons)
donorB (574 elements, 31 subgraphs, 272 singletons)
Occurence
(0.001, 0.1]
(0.1, 0.2]
(0.2, 0.3]
(0.3, 0.4]
(0.4, 0.5]
(0.5, 0.6]
(0.6, 0.7]
(0.7, 0.8]
(0.8, 0.9]
(0.9, 1.0]

D

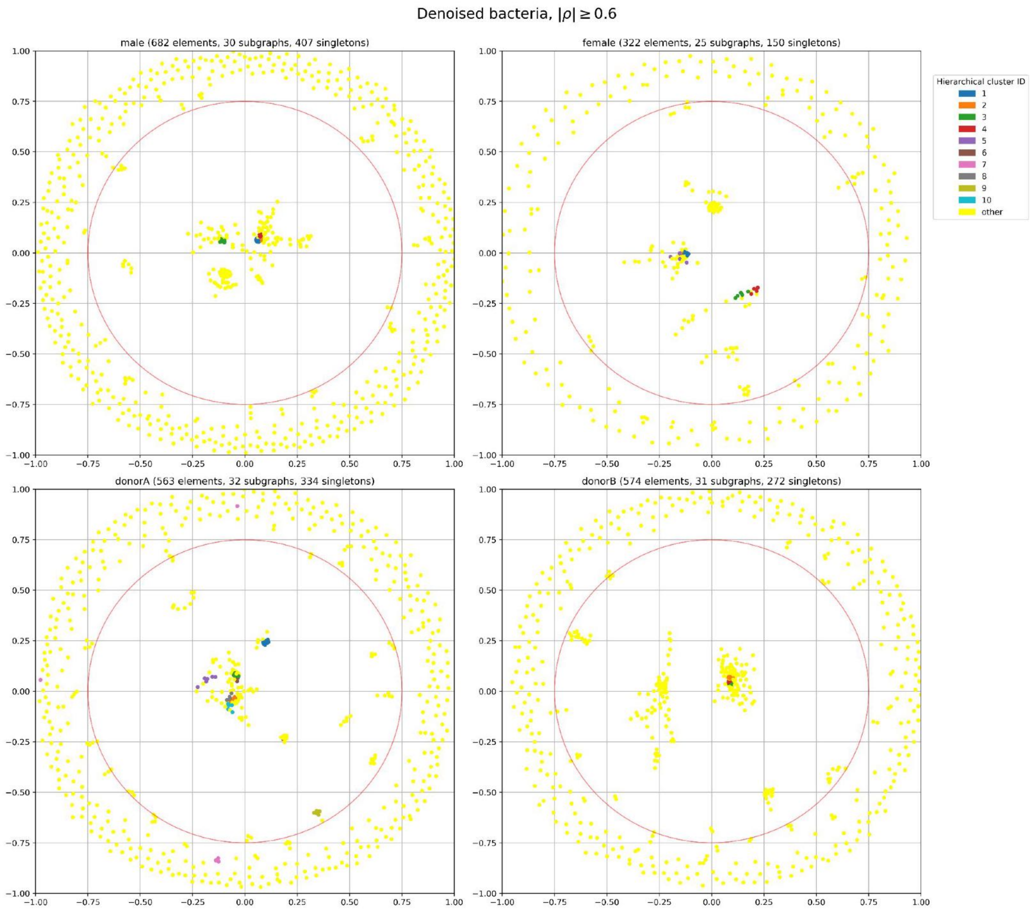
Denoised bacteria, $|\rho| \geq 0.6$
male (682 elements, 30 subgraphs, 407 singletons)
female (322 elements, 25 subgraphs, 150 singletons)
donorA (563 elements, 32 subgraphs, 334 singletons)
donorB (574 elements, 31 subgraphs, 272 singletons)
Hierarchical cluster ID
1
2
3
4
5
6
7
8
9
10
other

E

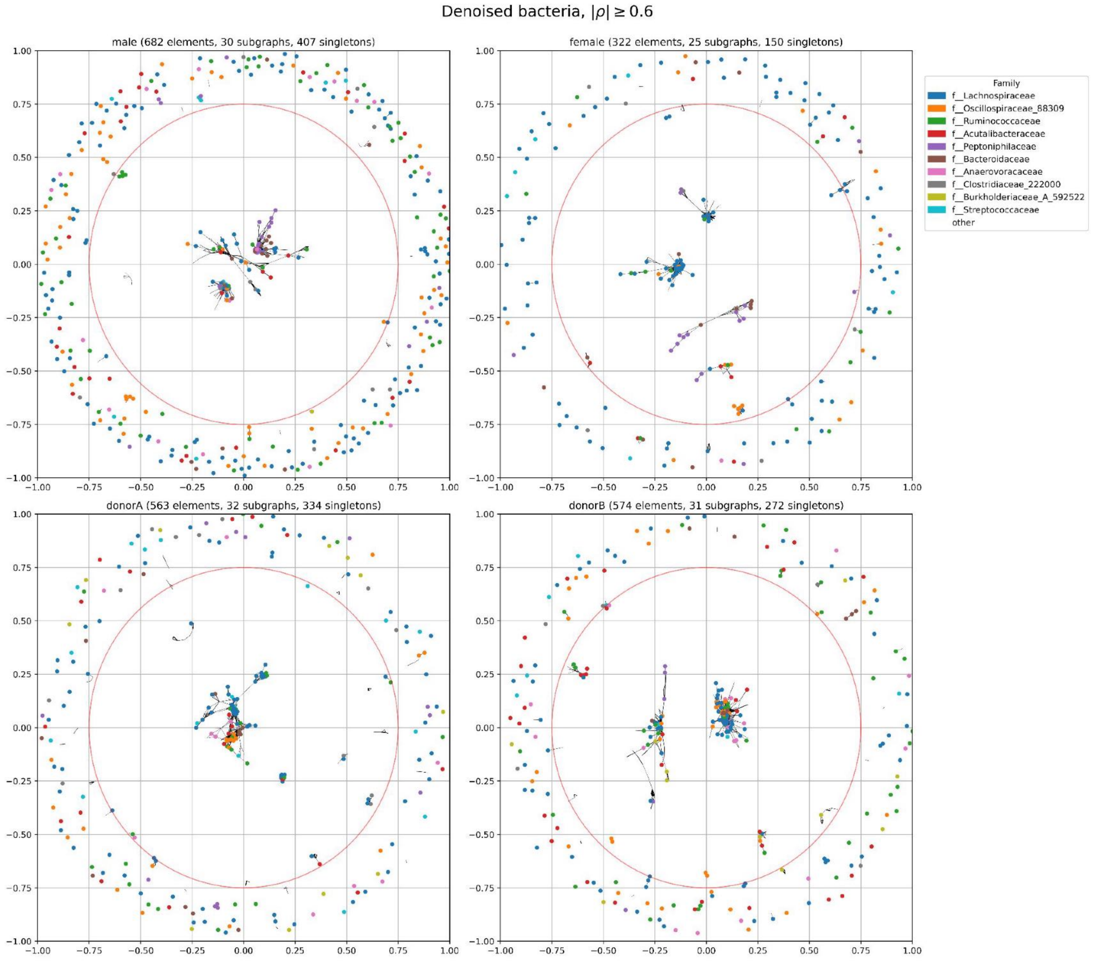
Denoised bacteria, $|\rho| \geq 0.6$
male (682 elements, 30 subgraphs, 407 singletons)
female (322 elements, 25 subgraphs, 150 singletons)
donorA (563 elements, 32 subgraphs, 334 singletons)
donorB (574 elements, 31 subgraphs, 272 singletons)
Family
f__Lachnospiraceae
f__Oscillospiraceae_88309
f__Ruminococcaceae
f__Acutalibacteraceae
f__Peptoniphilaceae
f__Bacteroidaceae
f__Anaerovoracaceae
f__Clostridiaceae_222000
f__Burkholderiaceae_A_592522
f__Streptococcaceae
other

F

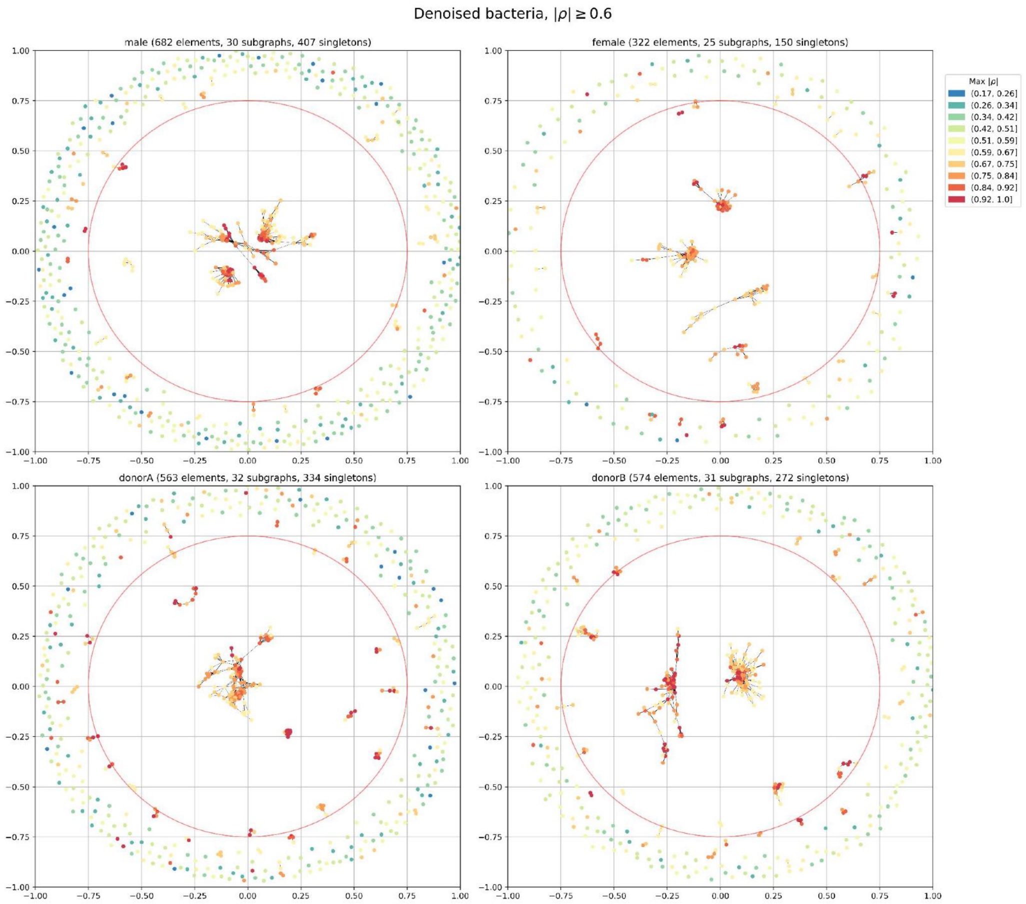
Denoised bacteria, |ρ| ≥ 0.6
male (682 elements, 30 subgraphs, 407 singletons)
female (322 elements, 25 subgraphs, 150 singletons)
donorA (563 elements, 32 subgraphs, 334 singletons)
donorB (574 elements, 31 subgraphs, 272 singletons)
Max |ρ|
(0.17, 0.26]
(0.26, 0.34]
(0.34, 0.42]
(0.42, 0.51]
(0.51, 0.59]
(0.59, 0.67]
(0.67, 0.75]
(0.75, 0.84]
(0.84, 0.92]
(0.92, 1.0]

G

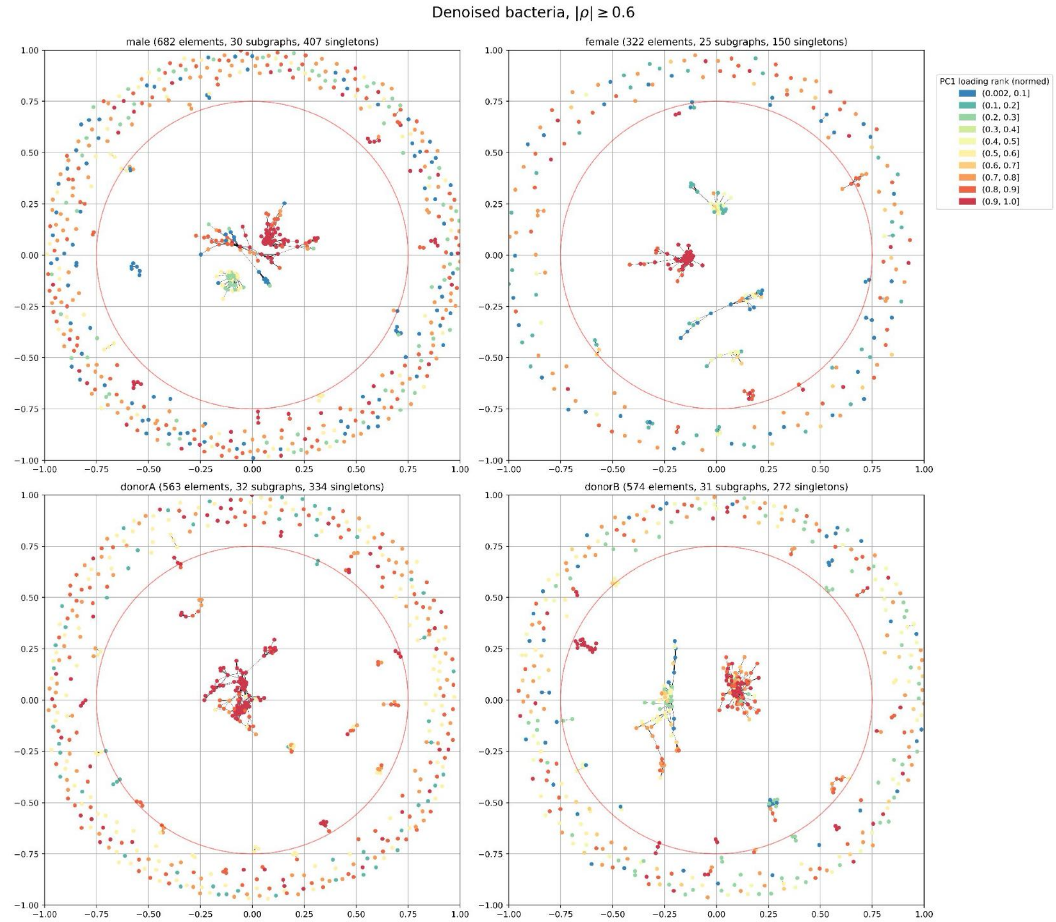
Denoised bacteria, |ρ| ≥ 0.6
male (682 elements, 30 subgraphs, 407 singletons)
female (322 elements, 25 subgraphs, 150 singletons)
donorA (563 elements, 32 subgraphs, 334 singletons)
donorB (574 elements, 31 subgraphs, 272 singletons)
PC1 loading rank (normed)
(0.002, 0.1]
(0.1, 0.2]
(0.2, 0.3]
(0.3, 0.4]
(0.4, 0.5]
(0.5, 0.6]
(0.6, 0.7]
(0.7, 0.8]
(0.8, 0.9]
(0.9, 1.0]

H

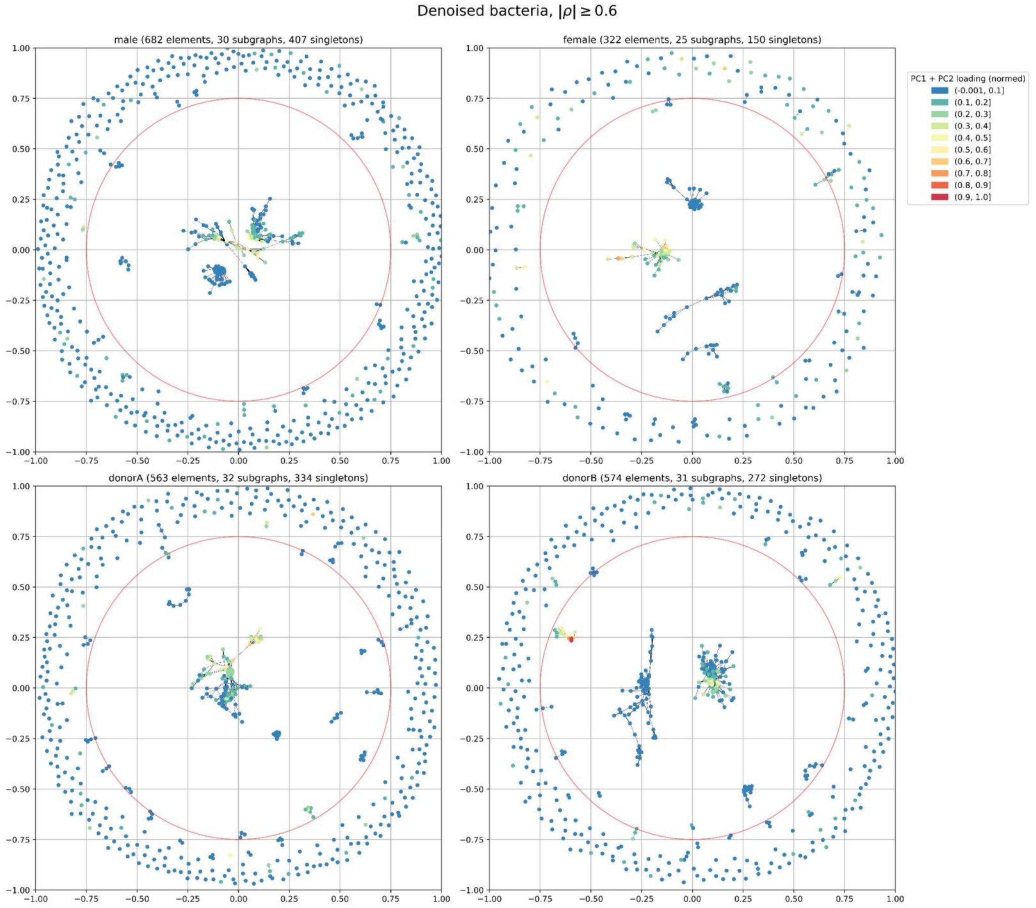

Denoised bacteria, |ρ| ≥ 0.6
male (682 elements, 30 subgraphs, 407 singletons)
female (322 elements, 25 subgraphs, 150 singletons)
donorA (563 elements, 32 subgraphs, 334 singletons)
donorB (574 elements, 31 subgraphs, 272 singletons)
PC1 + PC2 loading (normed)
(-0.001, 0.1]
(0.1, 0.2]
(0.2, 0.3]
(0.3, 0.4]
(0.4, 0.5]
(0.5, 0.6]
(0.6, 0.7]
(0.7, 0.8]
(0.8, 0.9]
(0.9, 1.0]

I

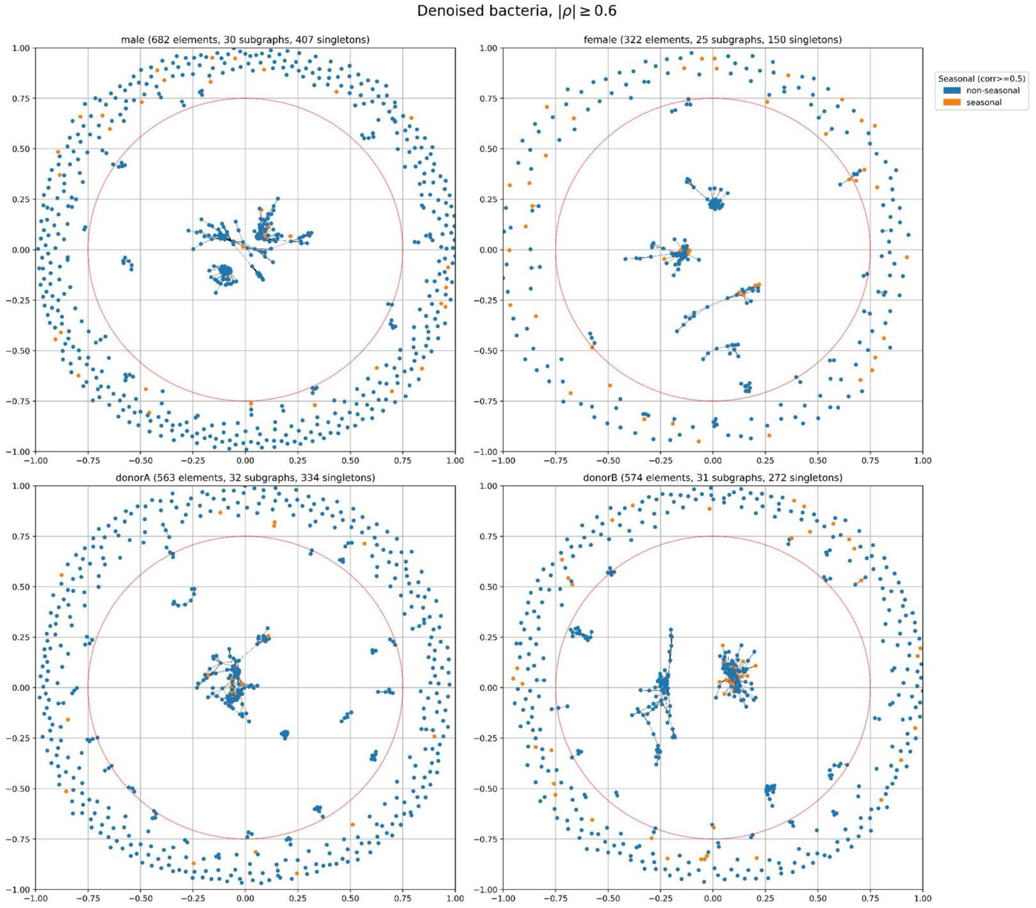

Denoised bacteria, |ρ| ≥ 0.6
male (682 elements, 30 subgraphs, 407 singletons)
female (322 elements, 25 subgraphs, 150 singletons)
donorA (563 elements, 32 subgraphs, 334 singletons)
donorB (574 elements, 31 subgraphs, 272 singletons)
Seasonal (corr>=0.5)
non-seasonal
seasonal

J

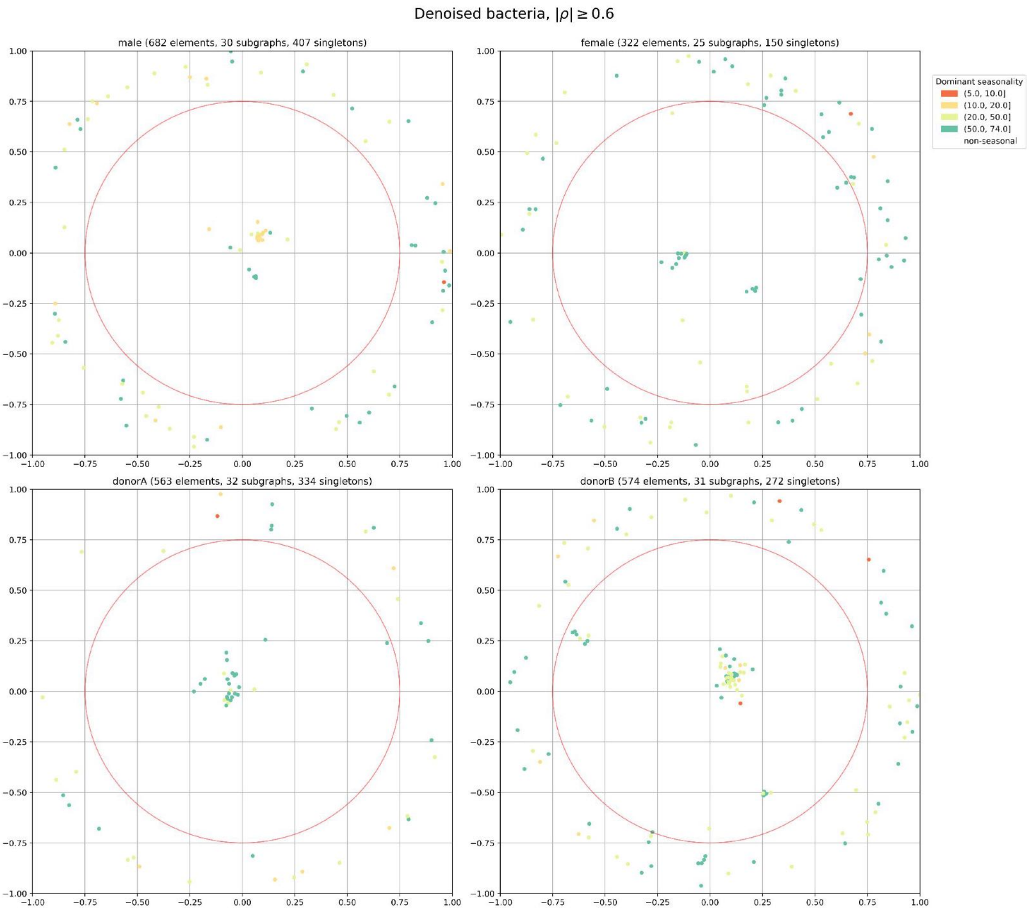

Denoised bacteria, $|\rho| \geq 0.6$
male (682 elements, 30 subgraphs, 407 singletons)
female (322 elements, 25 subgraphs, 150 singletons)
donorA (563 elements, 32 subgraphs, 334 singletons)
donorB (574 elements, 31 subgraphs, 272 singletons)
Dominant seasonality
(5.0, 10.0]
(10.0, 20.0]
(20.0, 50.0]
(50.0, 74.0]
non-seasonal

K

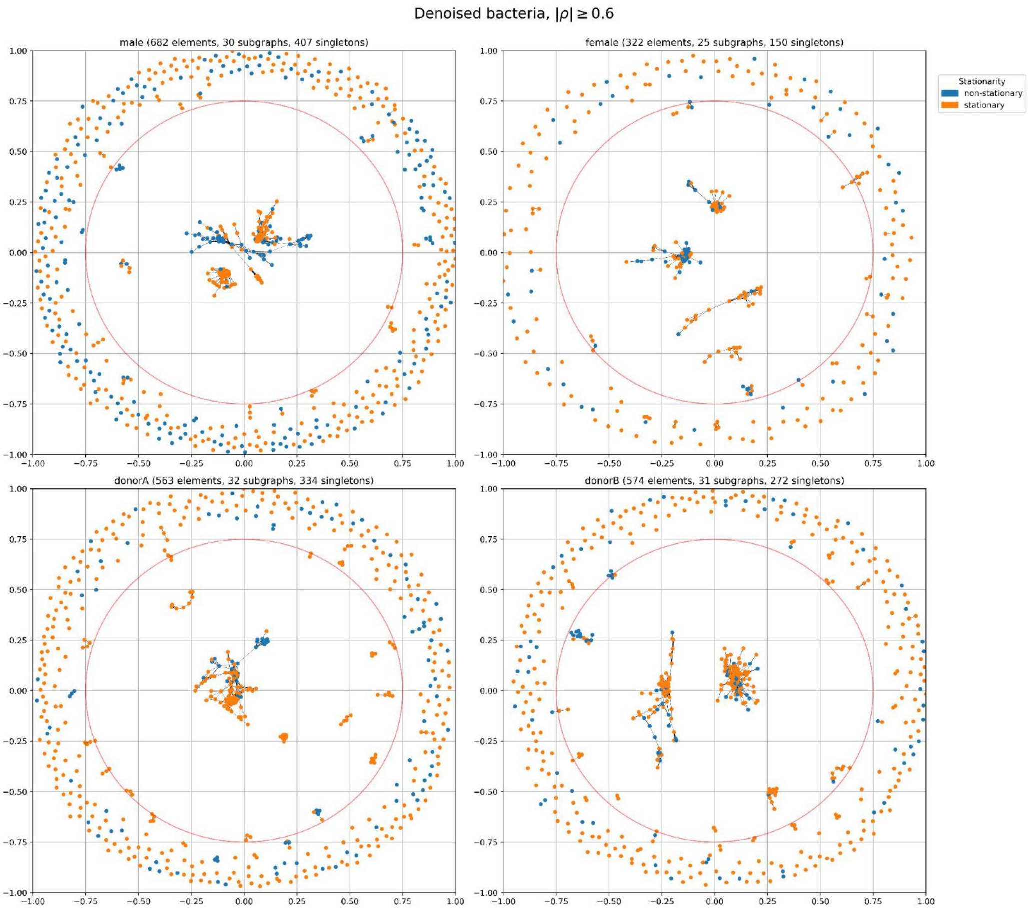

Denoised bacteria, $|\rho| \geq 0.6$
male (682 elements, 30 subgraphs, 407 singletons)
female (322 elements, 25 subgraphs, 150 singletons)
donorA (563 elements, 32 subgraphs, 334 singletons)
donorB (574 elements, 31 subgraphs, 272 singletons)
Stationarity
non-stationary
stationary

L

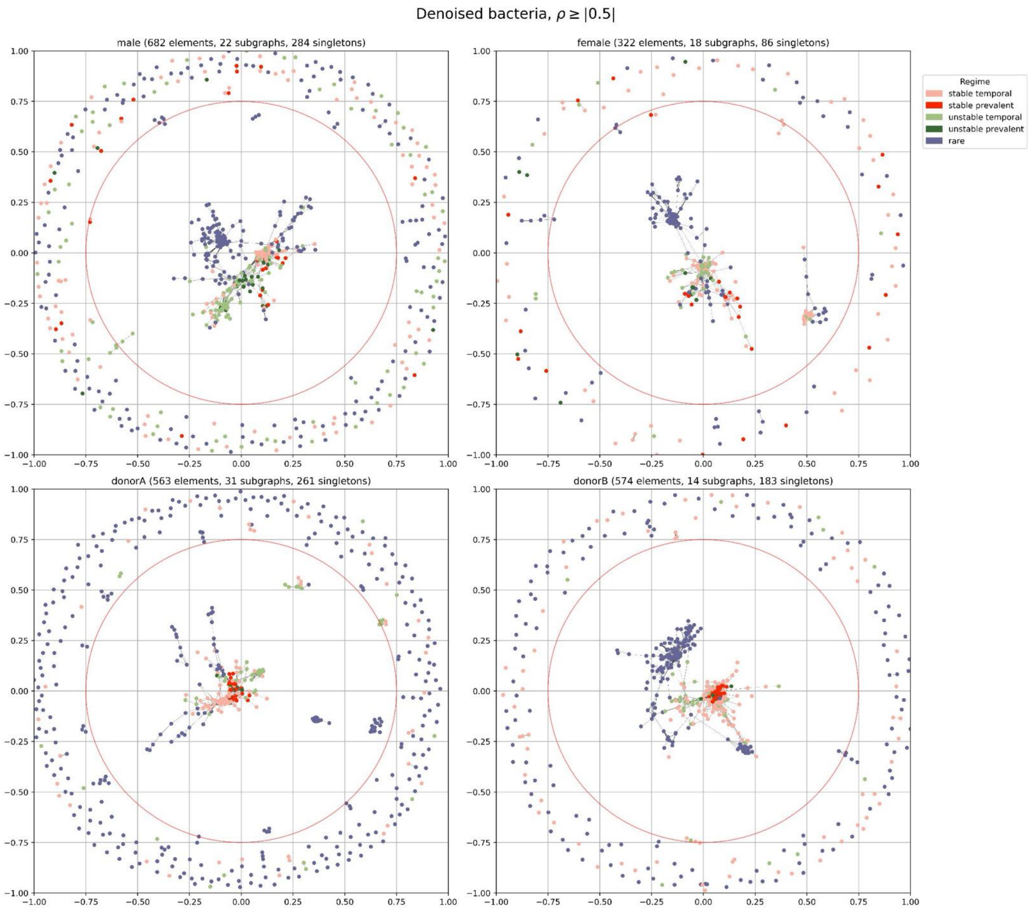
Denoised bacteria, $\rho \geq |0.5|$
male (682 elements, 22 subgraphs, 284 singletons)
female (322 elements, 18 subgraphs, 86 singletons)
donorA (563 elements, 31 subgraphs, 261 singletons)
donorB (574 elements, 14 subgraphs, 183 singletons)
Regime
stable temporal
stable prevalent
unstable temporal
unstable prevalent
rare

M

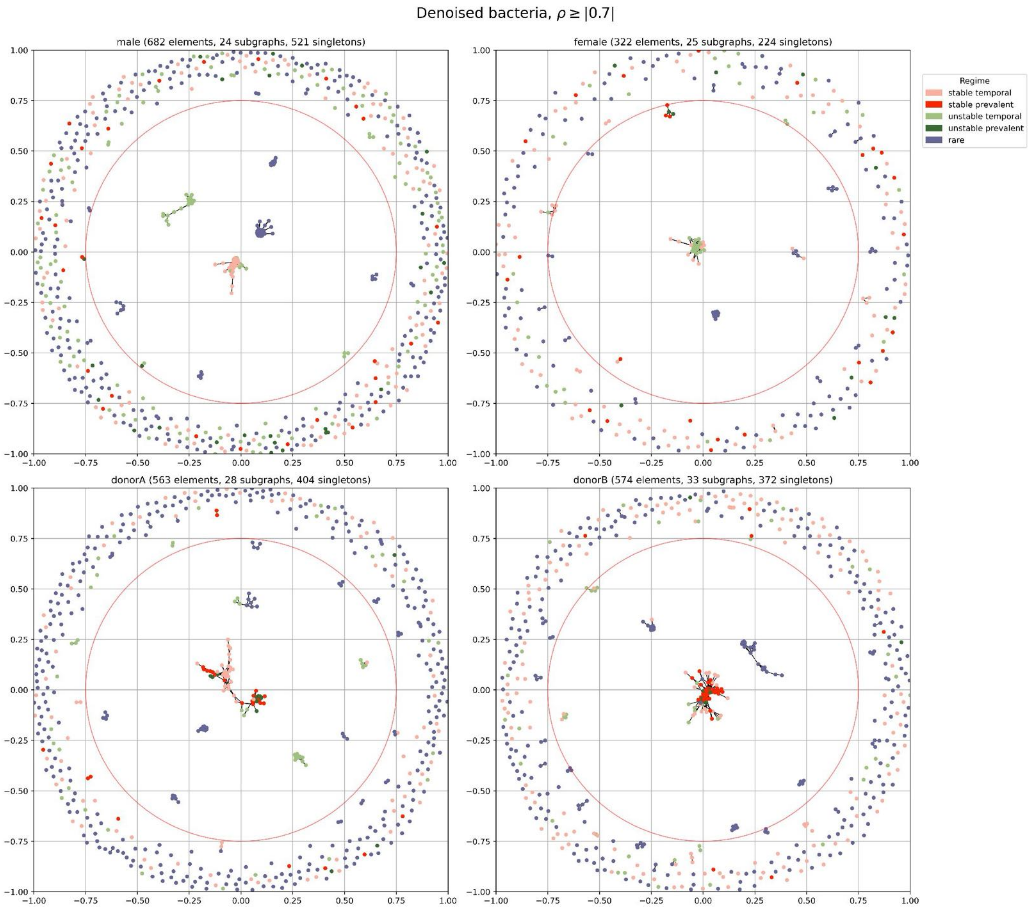
Denoised bacteria, $\rho \geq |0.7|$
male (682 elements, 24 subgraphs, 521 singletons)
female (322 elements, 25 subgraphs, 224 singletons)
donorA (563 elements, 28 subgraphs, 404 singletons)
donorB (574 elements, 33 subgraphs, 372 singletons)
Regime
stable temporal
stable prevalent
unstable temporal
unstable prevalent
rare

Supplementary Figure 15. Results of cluster analysis performed with NetworkX. Each subplot (**A-M**) represents a network (one panel per subject) of bacterial species (nodes) colored by different feature, where edges represent connections equal or stronger than ρ_{thr} = 0.6 (equivalent to $|\rho| \geq 0.6$) except subplot L and M, where ρ_{thr} = 0.5 and 0.7 have been used. In subplots A, B, L and M "none" represents bacteria that didn't pass rarefaction. Red circle in each panel separates the inner part from the cloud.

Regime evolution in time

Supplementary Figure 16 (see caption in p. 22) corresponds to Fig. 5B-C but for female and donorA subjects (panels A and B) or for male subject but generated using different ρ_{thr} (panels C, and D).

A

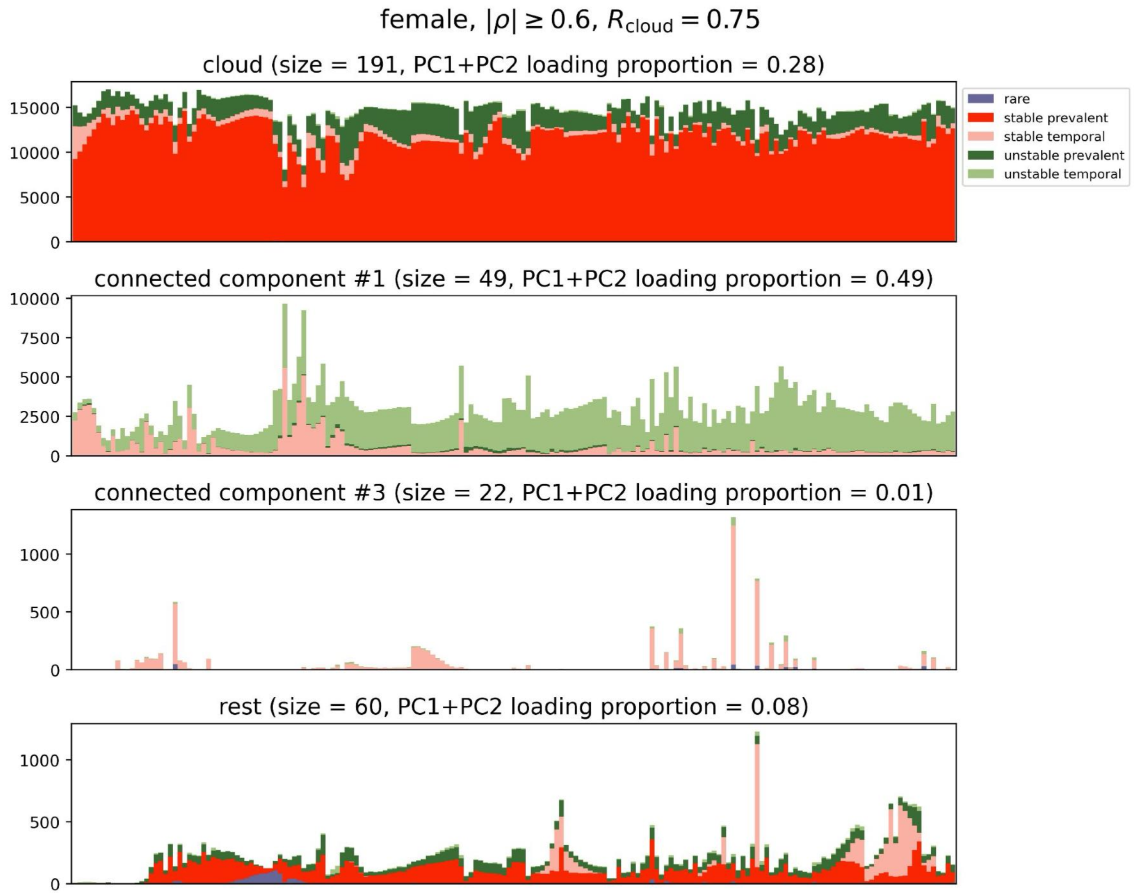

B

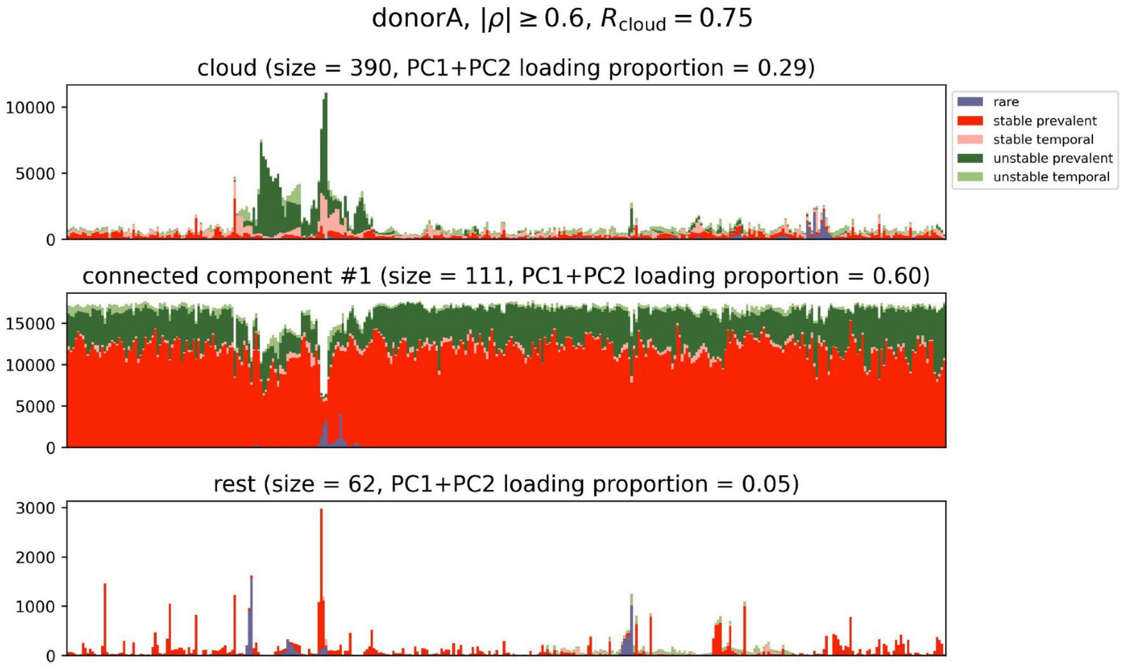
donorA, $|\rho| \geq 0.6$, $R_{\text{cloud}} = 0.75$
cloud (size = 390, PC1+PC2 loading proportion = 0.29)
10000
5000
0
rare
stable prevalent
stable temporal
unstable prevalent
unstable temporal
connected component #1 (size = 111, PC1+PC2 loading proportion = 0.60)
15000
10000
5000
0
rest (size = 62, PC1+PC2 loading proportion = 0.05)
3000
2000
1000
0

C

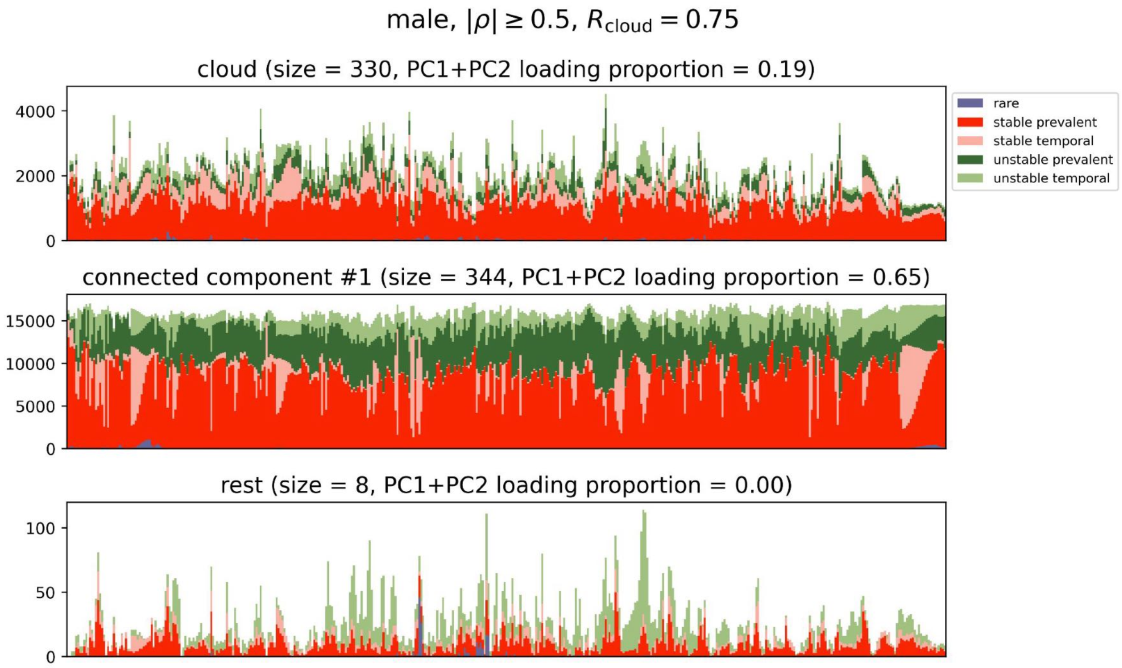
male, $|\rho| \geq 0.5$, $R_{\text{cloud}} = 0.75$
cloud (size = 330, PC1+PC2 loading proportion = 0.19)
4000
2000
0
rare
stable prevalent
stable temporal
unstable prevalent
unstable temporal
connected component #1 (size = 344, PC1+PC2 loading proportion = 0.65)
15000
10000
5000
0
rest (size = 8, PC1+PC2 loading proportion = 0.00)
100
50
0

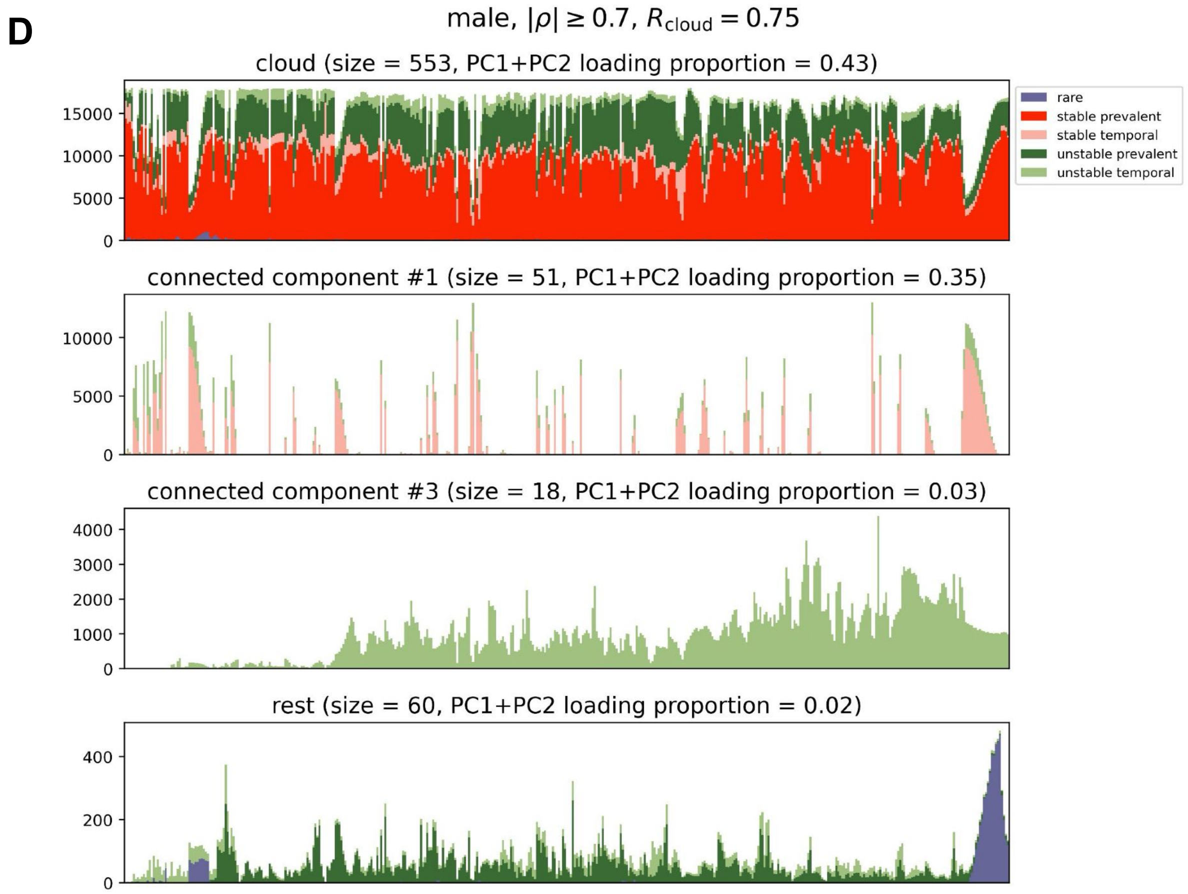

Supplementary Figure 16. Change in total counts after rarefaction over time for female (**A**), donorA (**B**) and male (**C, D**) subject stratified by regime (color) and group of bacteria (panels) i.e. cloud, largest connected components and the rest. In panel A and B ρ_{thr} = 0.6 whereas in panel C and D ρ_{thr} = 0.5 and 0.7 respectively. R_{cloud} represents a diameter that separates the inner part from the cloud.

Examples

Supplementary Figure 17 shows same example bacteria clustered together in the largest connected components. Heatmaps showing their mutual proportionality |ρ| are presented in Supplementary Fig. 18. Clearly, species placed close to each other on the graph have larger proportionality, which stays in line with the analysis presented in the next section (see Supplementary Fig. 19). Interestingly, in some cases (bacteria 3, 4, 5 versus 1, 2, 8, 9 in group A for female, bacteria 1 versus 2-6 in group A for donorA) we can notice anti co-occurrence, but in order to shed more light on the underlying effect behind it, both higher taxonomic resolution and functional analysis are required.

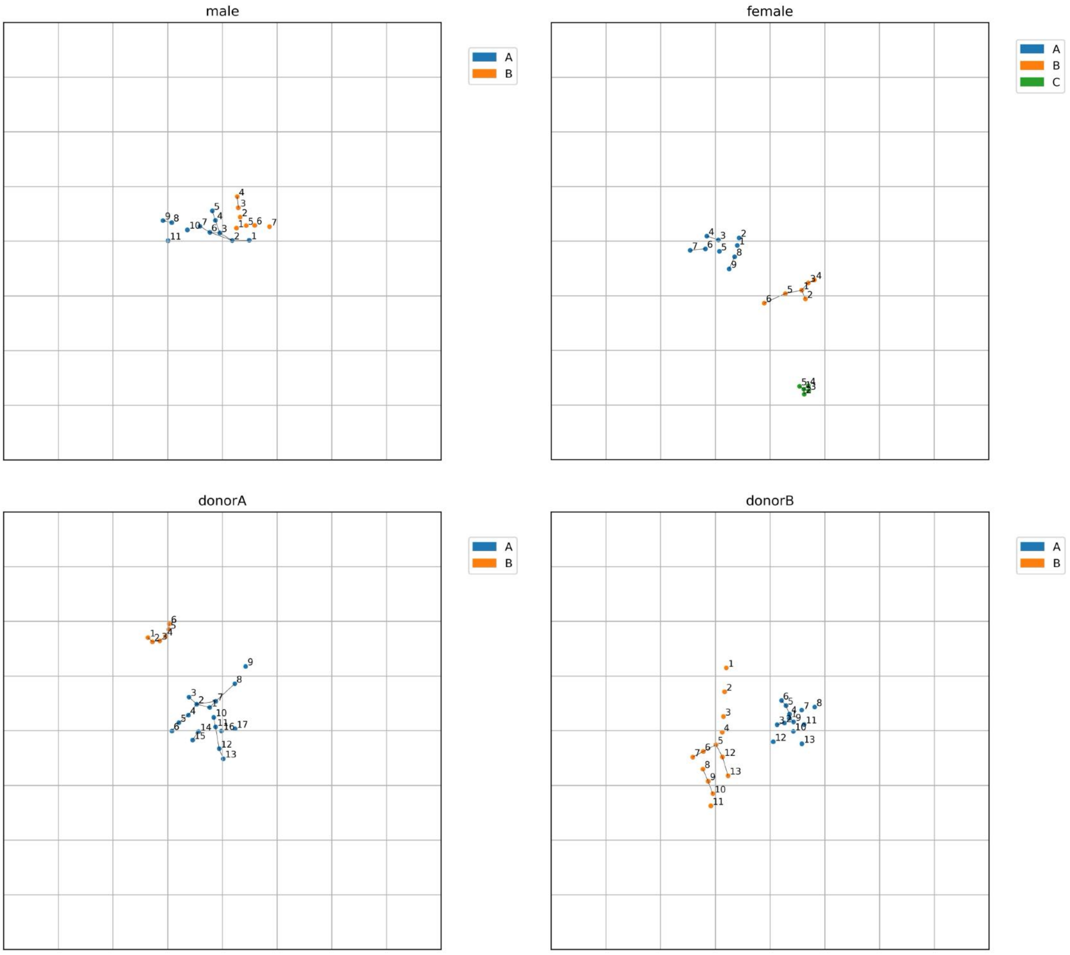

Supplementary Figure 17. Example bacteria selected from NetworkX graph shown in Fig. 5A in the manuscript. Each color for every subject represents a different group (cluster) of bacteria.

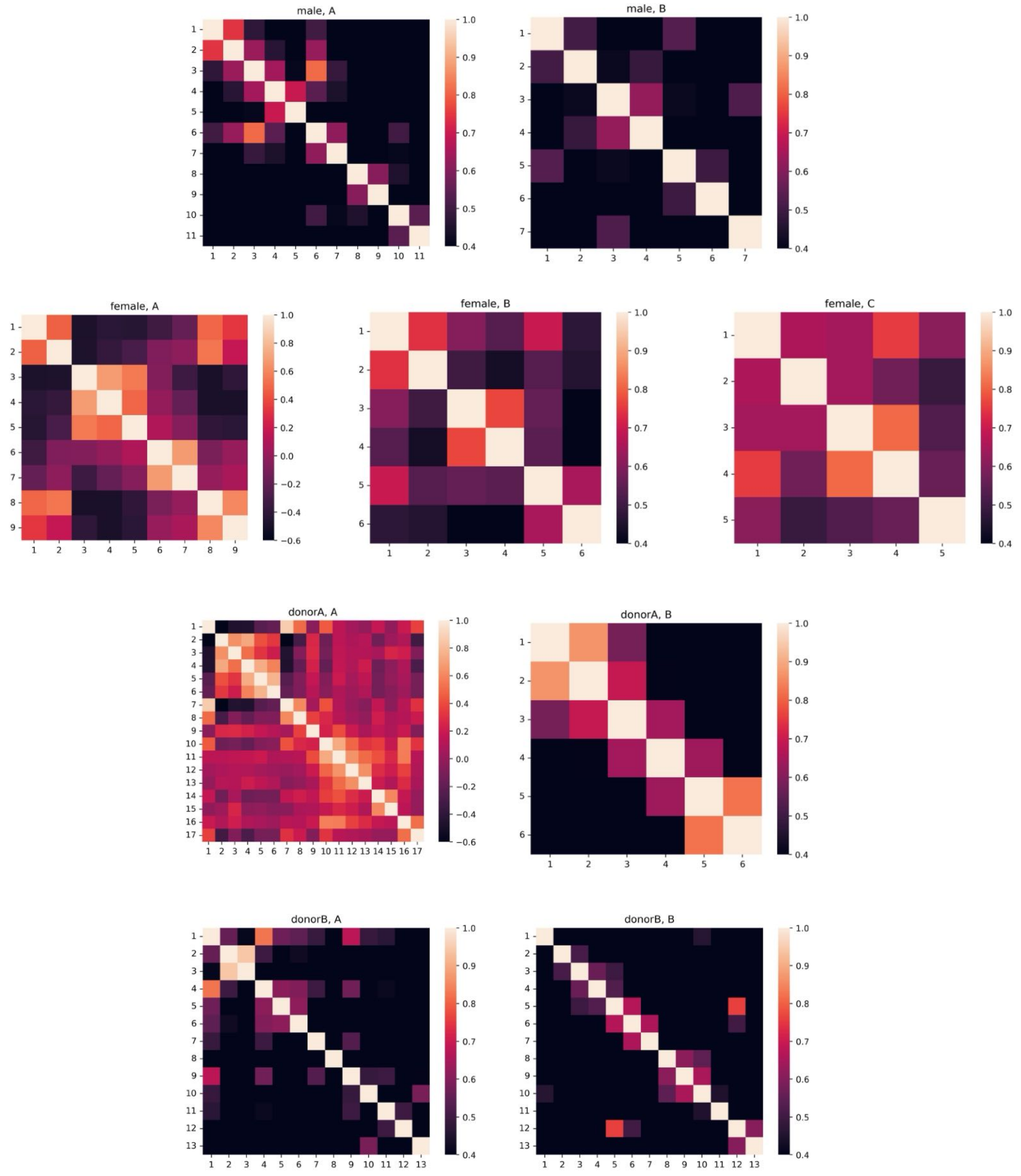

Supplementary Figure 18. Heatmaps showing proportionality between bacteria presented in Supplementary Figure 17 (one heatmap per bacterial group).

Sanity check - Euclidean distance vs $|\rho|$

In order to check whether similar bacteria are placed close to each other in the graphs we compared absolute value of their proportionality $|\rho|$ against Euclidean distance (Supplementary Figure 19). Indeed, we can see clear relation i.e. the larger $|\rho|$ the smaller distance which is evident especially for the largest connected components (left panels). When it comes to the cloud (right panels) existence of few points above the threshold ρ_{thr} (=0.6) comes from the fact that the cloud definition is imperfect and some smaller clusters were tiered apart by imposing the criterion on the cloud diameter value (R_{cloud} = 0.75). Distance distribution for $|\rho| \leq 0.6$ on the right panels shows that the polar angle doesn't matter for distant points (singletons in the cloud). However, this conclusion doesn't hold for the inner regions (left panels) which are of our special interest.

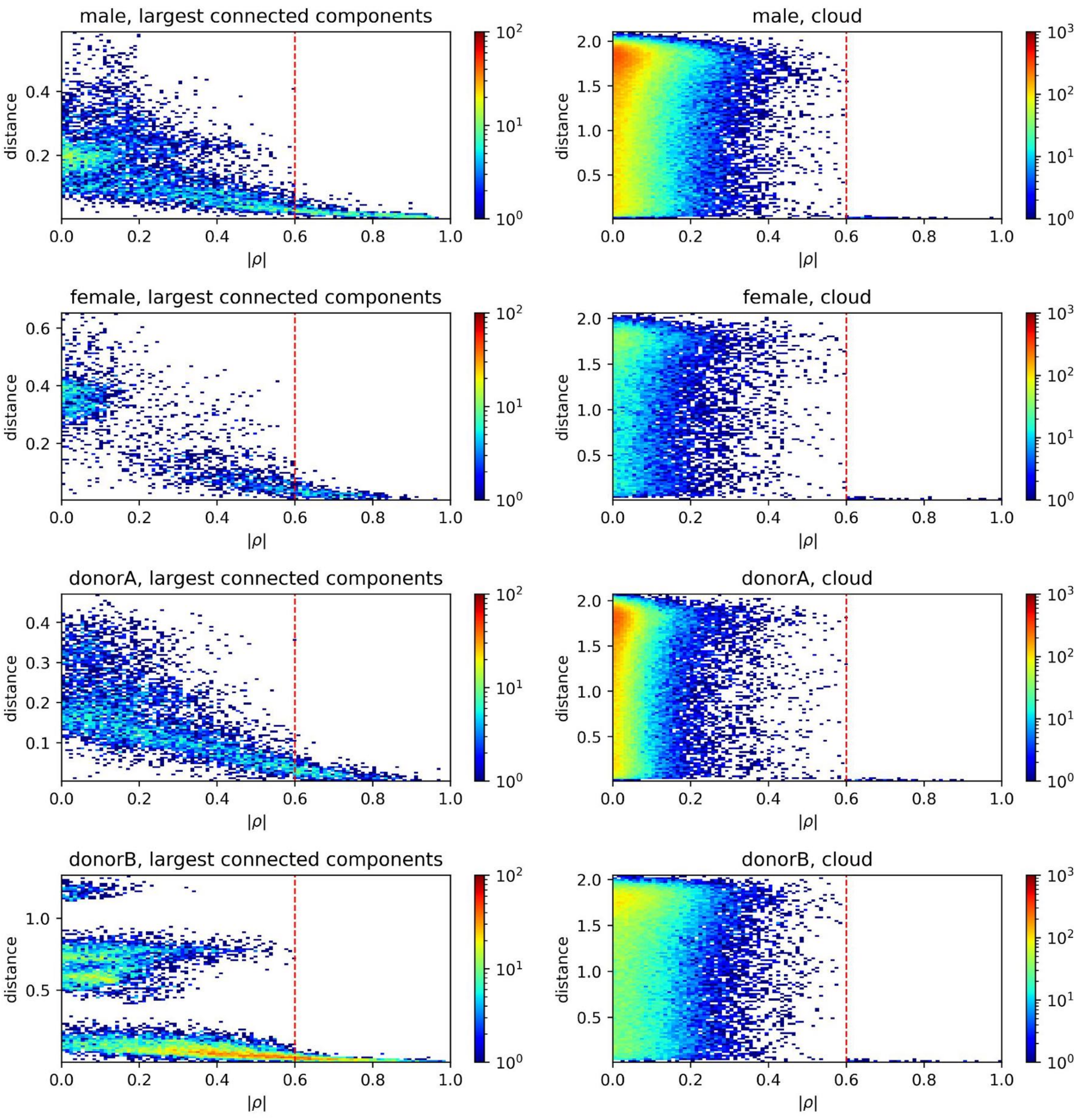

Supplementary Figure 19: Relationship between proportionality |ρ| and Euclidean distance of bacteria shown in NetworkX graph in Fig. 5 in the manuscript.

Hierarchical clustering

Using proportionality matrix as a feature matrix (treating it as a usual distance matrix didn't perform well) we constructed hierarchical clusters in order to compare them with the NetworkX graphs. As a sanity check we computed intra-cluster proportionality. According to expectations, the proportionality is high especially for the most abundant ones (see Supplementary Figure 20 and 21). Panel D in Supplementary Figure 15 shows location of the most abundant clusters (with cardinality ≥ 5 and mean abundance after rarefaction ≥ 0.1). Interestingly, they are mainly located in the central region of the graph (comprising largest connected components) supporting the hypothesis that this is the main part of the graph that drives the microbiome dynamics. We can also observe that some clusters e.g. 1, 2 and 4 for male subjects are placed very close to each other. Indeed, in this example the inter-cluster similarity is very high (ρ = 0.79 ± 0.12) and they can be treated as one larger cluster. It demonstrates that the NetworkX approach is more robust although it doesn't allow for strict cluster extraction.

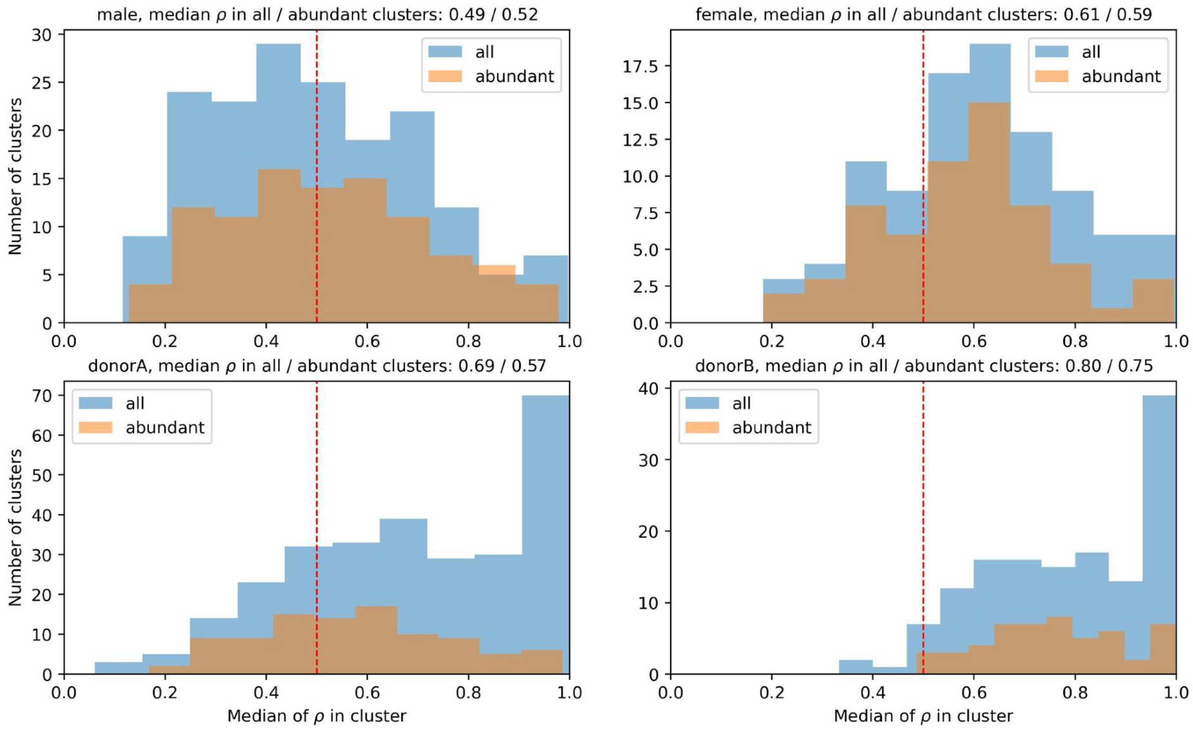

Supplementary Figure 20. Results of hierarchical clustering. Distribution of intra-cluster proportionality. Abundant clusters are clusters with mean bacterial abundance equal or higher than 0.1.

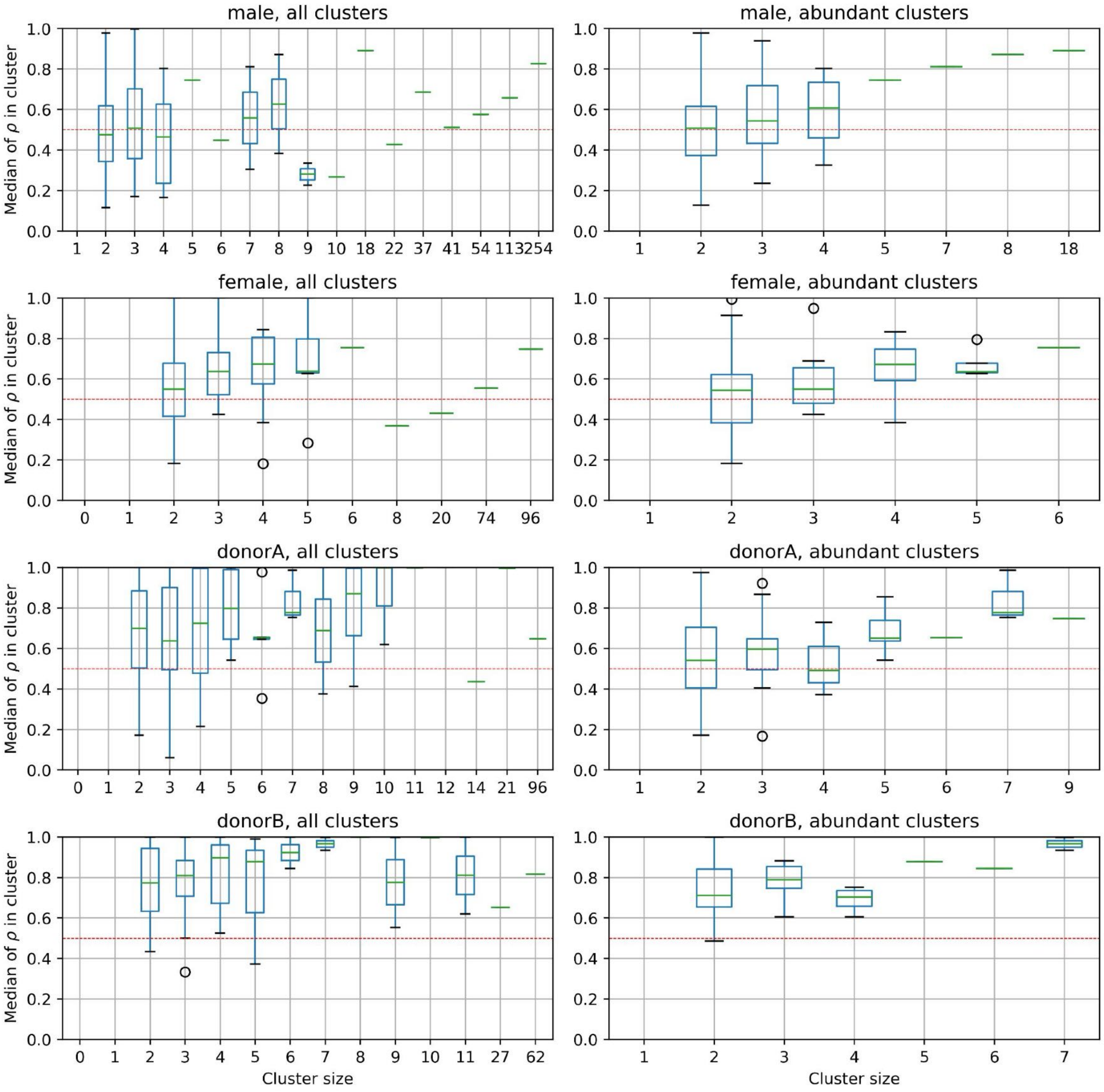

Supplementary Figure 21. The same as in Supplementary Figure 20 but each box plot corresponds to mean ρ distribution of bacteria in clusters of a given size (shown in x-axis).

Input data processing

Missing time points imputation

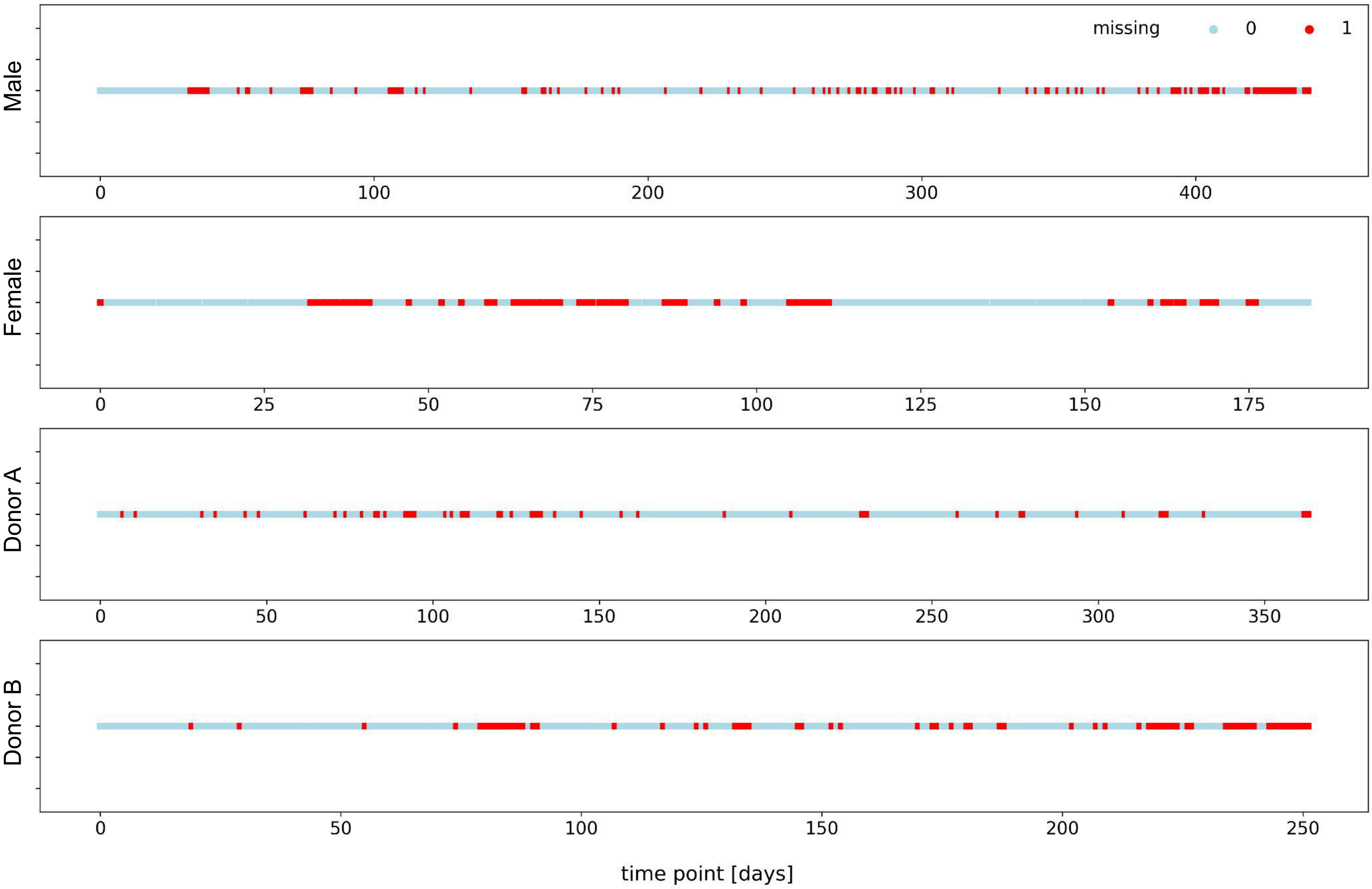

Supplementary Figure 22: Imputation of Missing Timepoints. Blue points indicate timepoints that were originally present in the dataset, while red dots signify timepoints that have been imputed using the PCHIP interpolation method

Input data processing

Rarefaction curves

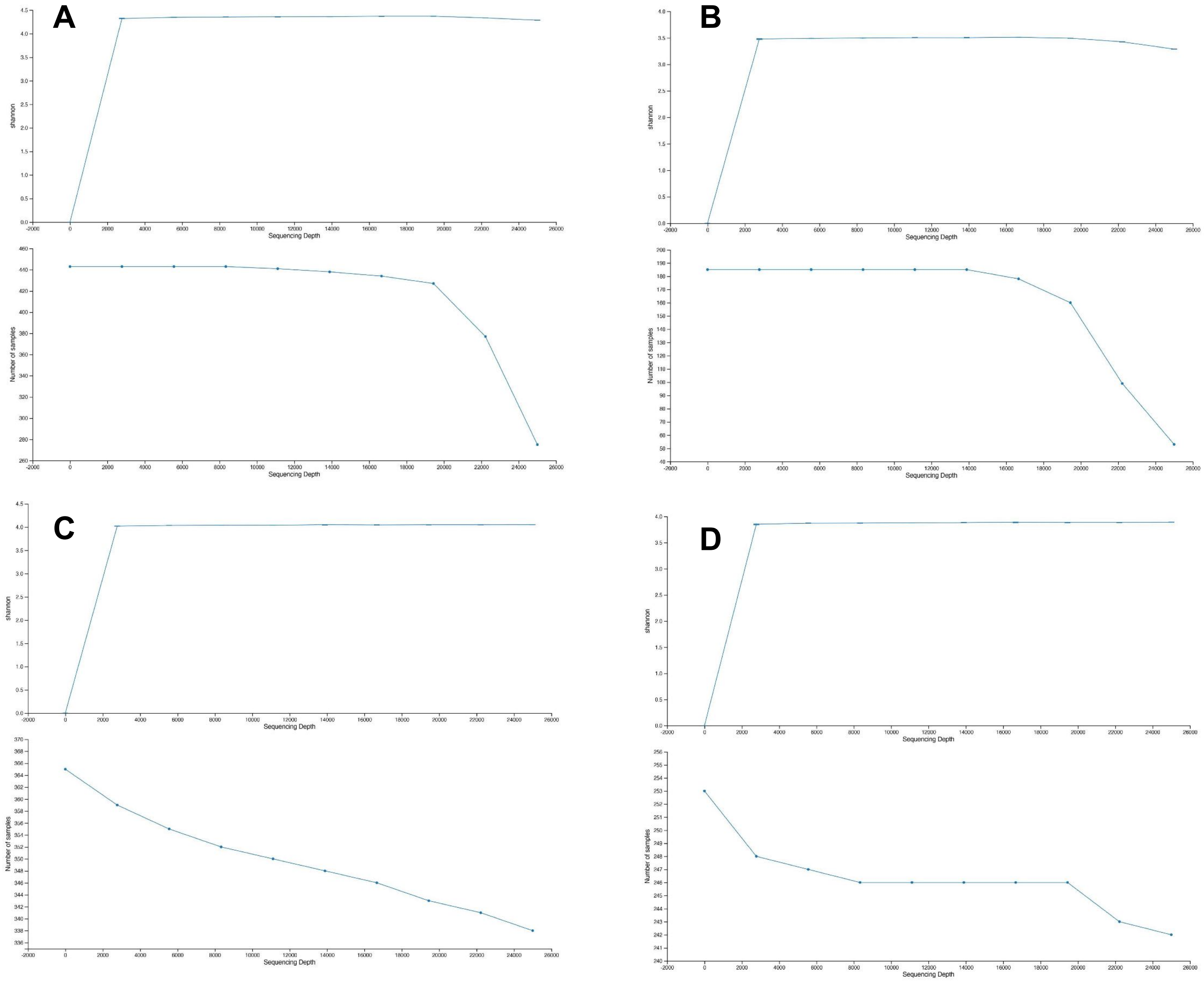

Supplementary Figure 23: Rarefaction Curves. For each subject, the upper plot illustrates the relationship between sequencing depth and the Shannon diversity measure. The lower plot depicts the relationship between sequencing depth and the number of samples meeting a specific threshold.
